# Racism and racial disparities in firearm violence: A scoping review

**DOI:** 10.1002/ajcp.70064

**Published:** 2026-04-16

**Authors:** Daniel B. Lee, Lexie M. Contreras, Laney Rupp, Stephanie H. Cook, Marc A. Zimmerman, Patrick M. Carter, Katherine P. Theall, Riley Bennett, Julia M. Fleckman

**Affiliations:** ^1^ Institute for Firearm Injury Prevention University of Michigan Ann Arbor Michigan USA; ^2^ Department of Social, Behavioral, and Population Sciences Tulane University School of Public Health and Tropical Medicine New Orleans Louisiana USA; ^3^ School of Public Health, Department of Health Behavior and Health Education University of Michigan Ann Arbor Michigan USA; ^4^ Social and Behavioral Sciences Department New York University New York New York USA; ^5^ Michigan Medicine Department of Emergency Medicine University of Michigan Ann Arbor Michigan USA; ^6^ Mailman School of Public Health Columbia University New York New York USA; ^7^ School of Public Health Washington University in St. Louis St. Louis Missouri USA

**Keywords:** firearm injury, firearm violence, racial health disparities, racism, scoping review

## Abstract

Firearm violence (i.e., interpersonal, police firearm violence) disproportionately affects racially minoritized communities. Researchers recently shifted their focus from race to racism to better understand the factors that contribute to racial disparities in firearm violence. Considering the emerging evidence base, we conducted a scoping review of published literature to: (1) summarize the quality of evidence in studies of racism and firearm violence, (2) identify key findings, and (3) prioritize steps for future research. A search for articles published between 1990 and 2025 across seven databases was conducted utilizing key terms. Of the 39 studies identified, 34 examined associations between racism and interpersonal firearm violence, and 5 examined associations between racism and police firearm violence. Residential racial segregation, historical redlining, racialized economic segregation, racial inequalities in socioeconomic outcomes, and other novel measures of racism (e.g., historical enslavement rate, the killing of George Floyd) were identified as risk factors for firearm violence. The majority of studies utilized cross‐sectional data, and a few studies examined mediators and moderators. Five future research priorities were identified, including: (1) developing a theoretical framework for the study of racism and racial disparities in firearm violence, (2) testing mechanisms between the relation between racism and firearm violence, (3) identifying protective factors, (4) applying a developmental perspective, and (5) improving data surveillance systems for firearm violence.

## Highlights

- Emerging research evidences the positive association between racism and firearm violence.
- Place‐based and cross‐sectional analyses used, with few mediators and moderators.
- Develop theoretical framework, test risk/resilience, use developmental lens, and enhance surveillance.

Interpersonal and police firearm violence is a public health issue in the United States (US; Center for Disease Control and Prevention [CDC], 2021). Akin to other public health issues in the US, non‐Hispanic Black individuals (Blacks) are 11.47 times more likely to die by firearm violence‐related deaths compared to non‐Hispanic White individuals (Whites; CDC, 2021). Further, while Blacks accounted for approximately 13% of the US population from 2001 to 2020, they accounted for nearly half (49.4%) of all non‐fatal firearm assault injuries. Hispanic and Indigenous/Native Americans individuals also face increased risk of firearm‐related injury and mortality relative to White individuals (CDC, 2021). Black, Indigenous/Native American, and Hispanic populations are also all significantly more likely to be fatally shot by police relative to White individuals. Racial disparities in police firearm violence are similar whether the victim is armed or unarmed (Sinyangwe et al., 2023).

As racial disparities in firearm violence have been consistently documented, there has been a recent shift towards examining racism as a contributor to these disparities (Bailey et al., 2017; Clark et al., 1999). This is because race is not a biologically meaningful construct, but rather a social construct developed in the 1700s to justify the enslavement of people from Africa (Trawalter et al., 2020). Racist ideology, therefore, led to the development of ‘race’ as a concept. Racism, broadly defined as “the exercise of power against a racial group defined as inferior by individuals and institutions with the intentional or unintentional support of the entire culture” (Jones, 1972, p. 112), is a multidimensional concept. Dr. Jones (1972) posited a tripartite model of racism. Individual racism (*hereafter*, interpersonal racism) manifests through interpersonal behaviors that maintain a power differential between racial groups. Institutional racism involves policies, practices, and programs that create differential access to resources and opportunities by race. Lastly, cultural racism encompasses intergenerational ideologies that reinforce beliefs in racial superiority or inferiority, perpetuated by media, language, and other socialization methods of the dominant group (Jones, 1972). Since the development of the tripartite model, other dimensions of racism, such as structural racism, have emerged in the literature (see Neblett EW, 2023). Structural racism refers to the totality of ways multiple institutions operate collectively to enact racially discriminatory policies (Bailey et al., 2017). Thus, recognizing its pervasive and pernicious influence across multiple dimensions (e.g., interpersonal, cultural), researchers have argued that racism, rather than race, is the fundamental driver of racial health disparities, including those in firearm violence.

A framework that guides the study of racism and racial health disparities, including racial disparities in firearm violence, includes the Framework for Study of Racism and Health (*hereafter*, FSRH; Williams & Mohammed, 2013). While other frameworks exist (e.g., Clark et al., 1999), the FSRH broadly identifies structural, institutional, and cultural racism as fundamental contributors to racial health disparities. The FSRH also delineates multilevel, multidomain pathways by which institutional and cultural racism deteriorate health for members of racially minoritized groups (Williams & Mohammed, 2013). For instance, due to the longstanding history of policies and practices that enforced residential racial segregation (e.g., redlining), historically segregated communities continue to have limited access to socioeconomic opportunities (e.g., upward socioeconomic mobility), societal resources (e.g., access to affordable housing and mental health care), and higher rates of racism‐related stressors (e.g., unfair treatment from doctors or police officers). These consequences, in turn, can contribute to firearm violence risk factors such as substance misuse (Cooper et al., 2016) and negative future expectations (Lee et al., 2020), which, according to separate studies, may influence firearm violence (Carter et al., 2015; Lee et al., 2020). To this end, historically redlined communities, compared to non‐redlined communities (e.g., Jacoby et al., 2018), and communities with higher levels of residential racial segregation (e.g., Knopov et al., 2019), tend to experience higher rates of firearm assaults.

The FSRH also suggests that racial disparities in police shootings result not only from individual officers' racial prejudice but also from a longstanding history of institutionalized control and violence against Black Americans. Modern policing originated in the 1700 s as “Slave Patrols,” which later evolved after the Civil War into forces that enforced Black Codes and eventually Jim Crow laws (Parks & Kirby, 2022). Police departments have long existed to uphold ideologies and practices reinforcing White supremacy. Owing to this longstanding history, one might surmise that even if overtly racist practices and ideologies are no longer explicitly endorsed, these acts of oppression may still be transmitted over time in subtler forms. The disproportionately high rate of fatal police shootings of unarmed Black victims relative to unarmed White victims is an example of the systemic biases within modern‐day policing. Even after controlling for the neighborhood crime rate, higher rates of fatal police shootings among unarmed victims have been observed in segregated, predominantly Black communities (Siegel et al., 2021). Researchers have, therefore, postulated that police departments may continue to view residents of segregated Black neighborhoods as inherently more threatening or criminal than residents of predominantly White neighborhoods.

Taken together, the FSRH posits that racial disparities in interpersonal and police firearm violence are due to ecological, social, and economic inequities generated by ongoing and historical forms of institutional and cultural racism. To our knowledge, the study of racism and firearm violence is still in its early stages but is growing rapidly and has the potential to inform interventions for addressing the root causes of racial disparities in firearm violence. We conducted a scoping review of published literature to examine the current evidence on the relationship between racism and both interpersonal and police firearm violence, and to broadly identify research gaps and future opportunities to better understand how racism contributes to racial disparities in firearm violence. The objectives of this scoping review are to (1) summarize the quality of evidence in the extant body of research on racism and both interpersonal and police firearm violence, (2) identify key findings, and (3) generate a series of future research priorities to guide the field.

## METHODS

We conducted this scoping review following Arksey and O'Malley's (2005) framework in three stages: sourcing articles, setting inclusion/exclusion criteria, and extracting data for result summarization.

### Search strategy

We worked with a librarian from the XXX to initiate the scoping review during the fall of 2022. Seven databases were searched: Medline, Scopus, Embase, Sociological Abstracts, Criminal Justice Abstracts, PsychINFO, and Cochrane. Search terms pertained to firearm violence and racism and were organized into Boolean search algorithms submitted to each database (see Supplementary Resource Appendix A). Search terms varied across databases, but generally included variations of firearm behaviors and enactments of racism across multiple levels (Jones, 1972). Firearm violence, defined by the CDC as any type of violence involving a firearm, encompasses homicide, non‐fatal assault, and unintentional injury. Our team expanded the definition of firearm violence, however, to include firearm‐related behaviors due to their increased risk of leading to injury. For example, we included firearm storage, carriage, ownership, and purchasing. The decision to include both firearm behaviors and injuries under the umbrella term “firearm violence” was made to ensure a comprehensive review, especially since this review is the first of its kind. Other firearm violence‐related search terms included, but were not limited to gun, gunshot, wound, carriage, purchase, discharge, revolver, and shotgun. With regard to racism, and guided by the tripartite model of racism (Jones, 1972), search terms included the enactment of institutional/structural racism (e.g., redlining, segregation), cultural racism (e.g., racist ideology, racial resentment), and interpersonal racism (e.g., racial mistreatment, microaggression). The searches were limited to English language articles focused on studies conducted within the US and published between 1990 and 2022. No additional articles were identified after expanding the search to 1980.

### Inclusion/exclusion criteria

Study abstracts were included if they reported findings from an empirical study examining the influence of racism on firearm violence in the US. Due to the Dickey Amendment, which prohibited the use of federal funds to “advocate or promote gun control,” firearm injury prevention research, including studies on racism and firearm violence, was drastically defunded (see Carter & Cunningham, 2016 for more details). Notably, this amendment, passed in 1996, prohibits the use of federal funds to advocate or promote gun control, effectively impeding research on firearm violence by restricting federal agencies (e.g., CDC) from funding or conducting research to address firearm violence as a public health issue. This has led to significant gaps in understanding and addressing the root causes of racial disparities in firearm violence. Firearm violence outcomes and risks were defined as: firearm access, storage, carriage, use, assault, victimization/injury crime, suicide, unintentional injury, police shootings, and the decision to shoot. Racism, the independent variable, included multiple forms of racism as indicated in the tripartite model of racism (Jones, 1972). This includes racially discriminatory actions from other people (e.g., racist hate crimes) to policies and practices embedded in key sectors of society, such as the government, labor market, the education system, healthcare, and media. In addition, we restricted our review to empirical research articles published in peer‐reviewed journals, excluding gray literature articles such as theses/dissertations, conference papers, and manuscripts not published in a peer‐reviewed journal. Moreover, gray literature (e.g., commentaries, editorials) was excluded for two reasons. First, our scoping review focused on empirical studies, which gray literature does not always encompass. Second, we only included studies that were peer‐reviewed to ensure the use of the best scientific methods and reduce the risk of including biased sources. Lastly, there were no exclusion criteria regarding study methodology or design (e.g., quantitative or qualitative studies).

### Article selection process

**Figure 1:**
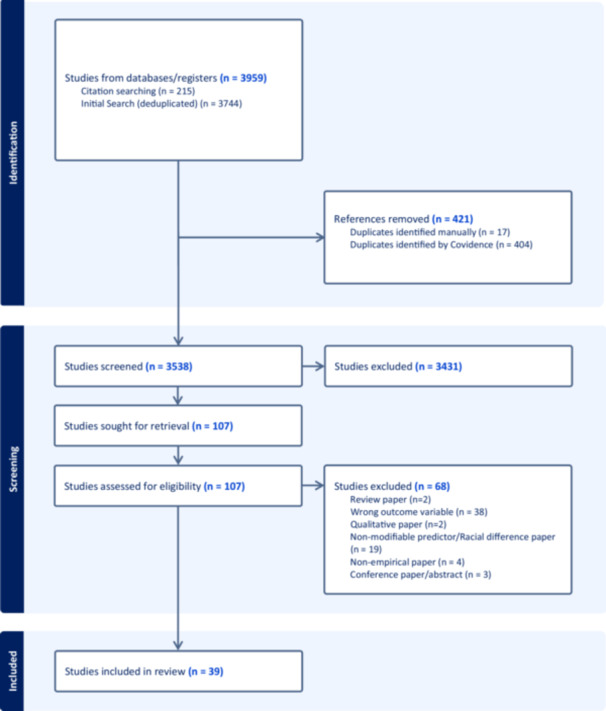
Systematic review PRISMA.

The team utilized Covidence software to manage and streamline our title and abstract review, and full‐text review (*Covidence Systematic Review Software*, n.d.). Figure 1 provides a visual representation of the article selection process. Our initial search yielded 3767 articles. Following Bramer and colleagues' deduplication model (Bramer et al., 2016), 1188 articles were removed in Endnote, leaving 2579 to be reviewed. Five reviewers independently evaluated all titles and abstracts to determine whether they met the inclusion/exclusion criteria. During this review, 2525 articles did not meet the inclusion criteria, and 54 potentially eligible articles were advanced to a full‐text review to either confirm inclusion criteria or provide additional clarification to finalize a decision. Further, we identified one qualitative study, but it did not advance to the full‐text review as racism was not conceptualized as a determinant of firearm violence. All articles advanced to the full‐text review were screened by two reviewers (independently and blindly) to assess eligibility, and consensus was met to determine exclusion factors. Discrepancies were resolved by a third reviewer. While inter‐rater reliability was not formally measured, discrepancies were rare due to the thorough training reviewers received in evaluating the methodological quality of studies. One review paper was identified that was relevant to our topic area. All citations from this review paper were imported into Covidence (133 articles), and two reviewers completed a second title and abstract review to identify any potentially eligible articles that were not captured by our original search. In this second importation, 15 duplicate title/abstracts were removed.

While conducting the full text review, the research team identified 15 laboratory‐based studies of racial bias. These studies were excluded for two reasons. Laboratory simulations may not accurately represent real‐world scenarios surrounding racism and firearm violence. The controlled environment and the lack of real consequences in a simulation could affect participants' behaviors, and findings may not be generalizable in a real‐world situation. Secondly, racial prejudice focuses on the perpetrator's attitude, whereas interpersonal, institutional, and cultural racism focus on victims' racism experiences. This decision resulted in advancing 39 studies to the data extraction and analysis stage.

### Data abstraction and assessment of methodological qualities

Data abstraction was completed by three faculty members, one doctoral student, and a research staff member. Reviewers were divided into two teams, and each team was randomly assigned articles. Reviewers completed a data template to organize findings and guide the abstraction. Variables included: author(s), year of study publication, setting, sample description including sample size, study design, theoretical models utilized (if any), firearm outcome(s) utilized, and types(s) of racism utilized and measurement, and relevant findings. In addition to conducting data abstraction, reviewers assessed the methodological quality of the included articles. Reviewers responded to 14 items for each study (see Supplementary Resource Appendix B) to determine if researchers used pre‐existing or novel theoretical models to situate their research questions, hypothesis, and interpretation of findings, sampling frame (e.g., neighborhood‐level vs. state‐level), measurement strategies (i.e., the utilization of validated measures to assess distinct dimensions of racism and firearm violence), data recency (i.e., years when measures were assessed), analytic approach (e.g., types of analyses conducted), mediators and/or moderators assessed, interpretation of results, and key limitations. Studies were reviewed by two individuals, with one faculty member participating in all review processes, and discrepancies were resolved by consensus. All articles and related abstracted information were reviewed by either the first author or senior author at completion.

## RESULTS

We conducted this scoping review to synthesize the current evidence on the relationship between racism and firearm violence. We utilized the FSRH to advocate for increased focus on racism as a key driver of racial disparities in public health issues, such as firearm violence (Williams & Mohammed, 2013). Accordingly, all studies identified in this scoping review contend that racial disparities in police and interpersonal firearm violence are rooted in institutional, cultural, and/or interpersonal racism, as outlined in the tripartite model of racism (Jones, 1972). As indicated in Table 1, almost all of the studies identified (38 out of 39) were published in 2017 or later. The 39 identified studies were sorted into two categories of firearm violence: interpersonal firearm violence (34 studies) and police firearm violence (5 studies). Findings from the quality assessment and the overview of results will be interpreted separately for interpersonal and police firearm violence. See Table 1 for specific study details related to methodological quality and key findings.

**Table 1:** Abstraction table of reviewed articles.

Authors and year	Study design	Location	Sample	Theoretical model used	Racism measurement	Outcome measurement	Racism and firearm outcome findings
Benns et al. (2020)	Cross Sectional	Louisville, KY	1307 GCVs residing in 310 neighborhood areas in Jefferson County (greater Louisville area)	Level of racism	Home Owners Loan Corporation (HOLC)	Gun Shot Victim (GSV)	Redlining associated with higher levels of GSV.
Buttrick & Mazen (2022)	Ecological, retrospective correlational	Multiple cities and states	Countries where people were historically enslaved in 1860 (Southern states)	Coping Model of Protective Gun Ownership	Historical enslavement rate; Racial segregation; Proportion free Black (1860); Black/White high‐school education ratio; Social connectedness index from Facebook	Percentage of suicides that are committed with a firearm, county level, 1999 to 2016. (Proxy for contemporary gun ownership levels.)	Rates of historical enslavement in a county and rates of contemporary gun ownership are positively correlated, after controlling for a wide array of county‐level covariates including residential racial segregation and voting patterns, supporting the hypothesis that a post‐Reconstruction cultural backlash is related to viewing guns as a measure of safety as opposed to danger. The ruggedness of a county (related to the “Culture of Honor” concept) is also correlated with gun ownership, but to a lesser degree. The association between historical enslavement and contemporary gun ownership is mediated by self‐reported feelings of being unsafe, largely restricted to counties that had slavery in 1860. Degree of social connectedness to counties with high rates of historical enslavement is also associated with gun ownership, which suggests that social attitudes spreading from the South to other areas of the country could partially explain gun ownership trends even in US counties where slavery was never legal.
Conrick et al. (2023)	Ecological, retrospective correlational	Multiple states	40 US states	N/A	Black‐white disparities in: Poverty, labour force participation, percent of individuals living in rental housing (all from ACS), educational attainment (National Center for Education Statistics), percent of single‐parent household (Diversity DataKids), arrests for index crimes (FBI UCR)	Disparity ratio of age‐adjusted rates of firearm homicide between White and Black populations by state. (source: WISQARS)	Absolute measures of disadvantage were inconsistently associated with Black‐White disparities in firearm homicide rates & percent black was not associated with firearm homicide rate at the state level. The disparity ratio for each measure of structural racism was always significant in predicting Black‐White disparities in the firearm homicide rate. Including the percent of population that is Black did not significantly improve the performance of the statistical model. This is evidence that it is racism, and not race alone, that leads to racial disparities in firearm violence experienced.
Dholakia et al. (2024)	Retrospective multilevel cohort study, correlational research	Multiple cities and states	8597 HOLC areas from 202 cities across 38 states, data from 2014‐2022	N/A	HOLC maps	Non‐suicide firearm fatalities	Between 2014 and 2022, 41,428 non‐suicide firearm fatalities were linked to HOLC areas. Across the 76,917 HOLC‐years, 15,693 (20.4%) contained at least 1 fatality. In both adjusted and unadjusted models, there was a dose‐response relationship between HOLC grade and firearm fatalities.
Ghio et al. (2023)	Ecological, retrospective correlational	Multiple cities and states	51 largest Metropolitan Statistical Areas (MSAs)	N/A	Index of Dissimilarity	Occurrence of Mass Shooting Events (MSEs)	The percent of the population in an MSA that is Black was positively correlated with MSE incidence, people injured per MSE, and people killed per MSE. Residential racial segregation only significant for spearman rho correlation, but not in linear regression. Percentage of Black is significant across the board (but percentage black is not a racism indicator).
Gobaud et al. (2024)	Longitudinal, retrospective correlational	Multiple cities and states	397 of the largest 500 cities in US (2010 census)	Critical Race Theory, Life Course Perspective	Structural racism measured in three different historical periods. HP 1 (1900‐1968): Presence of Redlining, Sundown town practices, Lynchings. HP 2 (1970‐2000): proportion of Black residents experiencing Unemployment, Jail, Owner occupied units, Poverty, No high school diploma. HP 3 (2010‐2019): Evidence of structural racism in Mortgage lending denial, Police killings, Arrests, Water pollution, Food desert, Per pupil revenue	Numbers and rates of interpersonal shootings (fatal, nonfatal, total)	All historical periods were correlated with all shootings, fatal shootings, and non‐fatal shootings. And each historical period predicts racism in subsequent historical periods
Hans et al. (2025)	Propensity‐score weighted difference in difference	Multiple cities and states	Exogenous population threshold whereby HOLC graded neighborhoods of US cities with populations of more than 40,000	N/A	Index of Dissimilarity; projected HOLC scores	Firearm fatality	There was an increase in overall firearm fatalities in treated cities; however, it was not statistically significant. There was a significant increase in fatalities occurring in low‐graded neighborhoods that were deemed risky for mortgage lending and a reduction in fatalities in high graded neighborhoods. The effect held consistently across various model specifications.
Hill et al. (2025)	Ecological, retrospective correlational	Kansas City, Kansas	Zip codes in Wyandotte County and Johnson County	Conceptual framework of Structural Racism Leading to Poor Outcomes	HOLC Redlining data	Counts of interpersonal firearm violence‐related injuries and deaths	Rates of interpersonal firearm violence injury were significantly higher in historically redlined communities compared to communities with historically better HOLC grades. Median income and percentage of poverty each also had univariate association with IFV injuries and deaths.
Houghton et al. (2021)	Cross Sectional	Multiple cities and states	51 largest metropolitan statistical areas (MSAs) with a Black population greater than 3% (as identified by William H. Frey) using data from 2013‐2017 ACS	N/A	Index of Dissimilarity	Firearm homicides	Residential racial segregation associated with firearm homicide.
Jacoby et al. (2018)	Cross Sectional	Philadelphia, PA	404 Census Tracts	Ecosocial approach, social disorganization theory	HOLC	Firearm assaults and homicides	Redlining associated with higher firearm assaults.
Knopov et al. (2019)	Retrospective	Multiple states	32 states over the period 1991‐ 2015	Ecosocial Theory of Racial Inequities in Health	Index of Dissimilarity	Black‐White Disparity in Firearm Homicides	Residential racial segregation associated with Black‐White firearm homicide disparity.
Krieger et al. (2017)	Cross Sectional	Massachusetts	Fatal and non‐fatal assaults for 1995‐2010 from MA Department of Public Health vital statistics and Weapons‐Related Injury Surveillance Systems (WRISS)	Ecosocial Theory of Distribution	Index of Concentrations at the Extreme (Race, Income, Racialized Economic); Index of Dissimilarity	Firearm Fatal Assault, Firearm Non‐Fatal Assault Injury	Racialized economic segregation was associated with higher rates of firearm homicides and non‐fatal assault injuries at census‐tract level. Index of dissimilarity was not associated with firearm homicides and non‐fatal assault injury at any level (census‐tract or city)
Larson et al. (2023)	Interrupted Time Series	Minneapolis, MN	Weekly firearm assault injury rates by ZCTA and period (pre‐killing, 0‐3 months post‐killing, 3+ months post‐killing)	N/A	Created time indicators that measure the average rate in the period as compared to the pre‐killing baseline. Key exposures: 1) the weekly linear time indicator, 2) an event indicator for the police killing of George Floyd on 5/25/2020 (post‐killing), and 3) a linear week time counter post‐killing.	Firearm assault injuries	Results showed a sharp but temporary surge in firearm assault injuries following George Floyd's killing, rising more than seven‐fold before returning to pre‐event levels. At the ZCTA level, firearm assault injury rates increased significantly in the three months after the killing, then declined steadily week by week. These patterns remained robust after accounting for seasonal variation and changes in police activity, indicating that the rise in gun violence was not explained by shifts in policing or COVID‐19 response.
Lee et al. (2024)	Cross‐sectional cohort study	Flint, Michigan	349 Black youth (ages 14‐24) from the Flint Youth Injury Study (2009‐2011) and 250 control recruited from an ER	Social disorganization theory	Index of Concentrations at the Extreme	Firearm aggression within 12‐24 months of baseline	The association between residential racial segregation and youth firearm aggression was not significant. However, residential racial segregation was positively associated with neighborhood disadvantage, neighborhood disadvantage was positively associated with exposure to violence, and exposure to violence was positively associated with youth firearm aggression. This shows evidence of an indirect pathway between residential racial segregation and youth firearm aggression. Specifically, residential racial segregation—as operationalized by ICErace – was indirectly associated with future youth firearm aggression by increasing neighborhood disadvantage and exposure to violence.
Lee et al. (2025)	Prospective cohort study	Flint, Michigan	349 drug‐using youth recruited from an emergency department as part of the Flint Youth Injury study and 250 controls also recruited from the same ED	Socioecological model, the code of the street theory	Index of Concentrations at the Extreme	Youth firearm carriage outside of home	The association between racialized economic segregation and firearm carriage was not significant. However, racialized economic segregation was positively associated with community violence, and community violence was positively associated with firearm carriage, leading to an indirect association between racialized economic segregation and youth firearm carriage. Those who carried firearms at wave 2, males, and those with higher retaliatory attitudes were more likely to carry firearms. Those exposed to community violence at wave 1 and younger youth were more likely to experience community violence.
Mehranbod et al. (2022)	Cross Sectional	Multiple cities and states	576 ZIP codes nested in 21 cities. Included ZIP codes were those for which data were available for historical redlining, historical demographic composition, present‐day violent and firearm death, and which had more than 10 ZIP codes per city.	N/A	HOLC; Index of Concentrations at the Extreme (Race)	Firearm Deaths	Redlining associated with higher levels of firearm death.
Ousey & Campbell Augustine (2001)	Cross Sectional	Multiple cities and states	109 cities that had 100,000 people and at least 3500 Black and 3500 White people	Strain theory, social control theory, social disorganization theory, and social isolation theory	Black‐White disparities in (1) income, (2) college graduation rate, (3) unemployment rate, and (4) isolation index (a measure of residential racial segregation) – aggregated	Firearm homicide offense rate (per 1,000) for Black youth and White youth (separate)	Structural racism index was not associated with firearm homicide perpetrated by White or Black youth (ages 14‐17)
Poulson et al. (2021)	Cross Sectional	Boston, MA	Firearm incidents (assaults and homicides) from 1/2016 to 12/2019; 7,530 census blocks were analyzed representing 180 census tracts	N/A	HOLC	Firearm assaults and homicides	Redlining associated with higher levels of firearm homicides and assaults. Association is mediated by socioeconomic status (poverty rate, poor educational attainment, and need for public services) and communities with larger Black population.
Poulson et al. (2023a)	CrossSectional	Boston, MA	Boston, MA (census block group for shooting rate & redlining; census tract for BIM and WIM)	N/A	HOLC	Firearm assault and homicides	Higher BIM is associated with greater reductions in firearm assaults/homicides for red and yellow desingated neighborhoods relative to green. Higher WIM is associated with higher firearm assaults/homicides for red and yellow designated neighborhood relative to green.
Poulson et al. (2023b)	Regression discontinuity	Boston, MA	City level data from January 2015 to December 2019	N/A	HOLC	Assault and homicides involving a firearm	A causal relationship between transitions from high or moderate harmful areas to desirable areas using the HOLC designations was found to be significant. Further, The authors showed the long term effects of redlining and its causal relationship to firearm incidence. Thus, when an areas transitions from a very or moderate harmful area to a desirable area firearm incidence decreases, but it increases the other way around.
Schleimer at al. (2022)	Longitudinal Analysis	Multiple cities and states	13 cities (zip codes within each city from 2018‐2020)	N/A	Index of Concentrations at the Extreme (Race, Income, Racialized Economic)	Firearm assault and homicides	Increases in firearm violence was significant larger in least privileged zip codes relative to the most privileged zip code (reference group).
Shour et al. (2023)	Retrospective correlational study	Wisconsin	72 Wisconsin counties	N/A	Index of Dissimilarity	Firearm fatalities	Counties with more segregation had more firearm fatalities compared to countries with less segregation. Counties with more income inequality had more firearm fatalities compared with countries with less income inequality. Overall conclusion, structural racism was associated with firearm fatalities across multiple counties in Wisconsin.
Siegal & Nicholson‐Robinson (2025)	Retrospective correlational study	Multiple cities and states	National sample of 104 counties using census‐tract, county‐level and school data from 1990‐2020	Life course perspective, cumulative disadvantage theory	Index of Dissimilarity; Entropy Index	Racial disparities in total firearm homicide rates	Residential racial segregation was largely unchanged from 1991‐2020 (index of dissimilarity from 0.40 to 0.41). School racial segregation increased slightly from 1991‐2020 (0.36 to 0.39) with most of the increase happening between 1991‐2000. Counties were then grouped into 3 categories based on segregation and increase in segregation from 1991‐2020 (1 being lowest, 3 being highest). The rate of racial disparities in firearm homicide rate per 100,000 people during the total study time period was dependent on segregation grouping (3.97 to 5.51 to 8.77). Counties with higher segregation also saw greater increases in disparities between 1990‐2020. Higher levels of baseline school segregation in 1991 was a significant predictor of the likelihood a county has higher disparities in firearm homicide rates. After controlling for baseline school segregation, increase in school segregation was also a significant predictor of higher disparities in firearm homicide rates. Both baseline residential and school segregation were significant predictors of the black‐white firearm homicide rate ratio from 2002‐2017.
Siegel & Wiklund (2023)	Retrospective correlational study	Multiple cities and states	44 states; state‐level data from 1999‐2020	N/A	Structural racism, operationalized using a scale consisting of measures of segregation, and black‐to‐white ratios of incarceration, economic status/wealth, employment, and education	Firearm homicide rates, operationalized as age‐adjusted homicide rate by county disaggregated by race, from CDC WISQARS	43% of the variance of black‐white firearm homicide rates was attributed to the county structural racism score. Using linear regression, each one standard deviation increases in structural racism score increased the firearm homicide rate ratio by 1.60 (95% CI: 1.52, 1.70). Inclusion of the control variables did not significantly alter the relationship between structural racism score and firearm homicide rate ratio.
Siegel et al. (2023a)	Cross‐sectional, retrospective correlational study	Multiple cities and states	44 states; state‐level data	Krieger's ecosocial theory	Black/White disparity ratios for Incarceration rates*, Residential segregation (Dissimilarity Index at Census tract level*, Isolation Index, Entropy Index, Divergence Index, Index of Concentration of Extremes (ICE) for Racialized Economic Segregation), Economic Status/employment (Income, Rental, Poverty, Non‐labor participation, Racial opportunity gap, Unemployment rate*, managerial occupation, service occupation), Education (No high school degree*, no college degree), Political participation and representation (Voting participation, Felony disenfranchisement*, State legislative representation), Environmental Racism (RSEI disparity, Environmental burden score, Particulate exposure, Fine particulate matter air pollution*), Racial Equity Index/Inclusion Score*/Prosperity Score. Measures with asterisk (*) appeared in final model.	Non‐Hispanic Black‐White disparities in death rates for firearm homicide, by state. (source: CDC WISQARS database) (other study outcomes not related to firearms)	There was a statistically significant positive relationship between the level of structural racism in a state and the magnitude of the Black‐White racial disparity for firearm homicide. Associations were detected also between their structural racism measure and HIV, infant mortality, obesity, and asthma. (Also included was a state‐by‐state map of the final structural racism measure they constructed.)
Siegel et al. (2023b)	Retrospective correlational study	Multiple cities and states	395 US counties, data from 2016‐2020	Ecosocial Theory, Conflict Theory, Race Stratification Theory	Structural racism, operationalized using a scale consisting of measures of segregation, and black‐to‐white ratios of incarceration, economic status/wealth, employment, and education	Firearm homicide rates obtained from CDC WISQARS from 1999‐2020	Across the states measured, the average ratio of black‐to‐white mortality from firearm homicide was 10.7 (log scale), which was statistically significant at the 95% confidence level. Each 1 point increase on the structural racism scale was associated with a 65% increase in the ratio of black‐to‐white firearm homicide rates (95% CI: 0.48‐0.84). Structural racism explained 67% of the variation in the observed racial disparity in firearm homicide rates across states.
Siegel et al. (2024)	Ecological, Cross‐sectional, Retrospective correlational	Multiple cities and states	776 US cities; city‐level data	Conflict theory, Race stratification theory	Structural racism measured with Standardized Structural Racism Factor Score. Constructed with Black‐White disparity ratios in five different dimensions: Incarceration (Local Jailing Rate*), Segregation (Dissimilarity index*, Isolation Index, Entropy Index, ICE for Racialized Economic Segregation), Economic (Median Income*, Poverty rate*, Rental, non‐labor participation), Education (no high school degree*, no college degree), Employment (Unemployment, Managerial Worker*) (many sourced from ACS data). Measures with asterisk (*) appeared in final model.	Black‐White disparities in firearm homicide rates	The structural racism factor score was significantly associated with the racial disparity in firearm homicide (n=315). About 6% of the variation in the difference in magnitude in racial disparity in firearm homicide rates across cities was explained by the city structural racism factor score. A color‐coded U.S. map was made to display the standardized structural racism factor scores for 776 cities.
Spitzer et al. (2006)	Retrospective cohort study	Multiple cities and states	Nationwide (USA) ‐ ZCTAs	N/A	HOLC	Incidence of firearm injury	When controlling for urban firearm risk factors, neighborhoods with detrimental redlining were associated with 2.6 additional firearmincidents annually compared with non‐redlined areas with similar modern‐ day risk factors. This resulted in an additional 23,000 firearm injuries over the study period.
Su et al. (2024)	Cross sectional	Multiple cities and states	2,584 individuals (oversampled racial and ethnic minority groups)	N/A	Self‐reported direct experience of racism in 2020/during COVID‐19 pandemic, sourced from Health, Ethnicity, and Pandemic (HEAP) Survey.	Self‐reported Firearm purchasing	Self‐reported experience of race‐related hate crimes during the pandemic was significantly associated with purchasing a gun during the pandemic, in both unadjusted and adjusted models.
Uzzi et al. (2022)	Cross Sectional	Baltimore, MD	3435 non‐fatal shootings from 2015‐2019	Intersectionality Theory	HOLC; Index of Concentrations at the Extreme (Racialized Economic Segregation)	Firearm non‐fatal assaults	Sustained disadvantaged (red/yellow lined + higher median in ICE) associated with higher rates of non‐fatal firearm assault injuries
Uzzi et al. (2024)	Ecological Cross Sectional	Baltimore City, Maryland	146 census tracts of Baltimore City, MD	The goal of article is to present a novel theoretical framework that offers a conceptualization of the relationship between racial capitalism and firearm violence within the context of neighborhoods in the US.	HOLC; tract‐level ICE scores from Home Mortgage Disclosure Act database 2004‐2006	Nonfatal and fatal shootings	Sustained disadvantaged census tracts (tracts that were historically redlined and experienced higher contemporary subprime lending) experienced the highest burden of firearm violence in Baltimore City between 2015 and 2019.
Wong et al. (2020)	Longitudinal Analysis (18 years, 2000‐2017)	Multiple cities and states	275 Cities	Contact hypothesis	Index of Dissimilarity; Black‐White disparities in 8 (1) unemployment rate, (2) labor force nonparticipation rate, (3) poverty rate, (4) renting rate, (5) single parent‐households, (6) persons without a college degree, (7) median household income, and (8) incarceration rate.	Black‐White ratio of firearm homicide rate; Black firearm homicide rate; White firearm homicide rate	Residential racial segregation (index of dissimilarity) predicts classification into trajectories with a higher levels of Black‐White disparity in firearm homicide rates. Higher levels of residential segregation was associated with classifying into a trajectory with higher levels of Black and White firearm homicide rates (trajectories for each race). Lastly, per the multilevel model (cities nested within states), residential segregation AND Black‐White disparity in poverty rate was associated with higher Black‐White disparity in firearm homicide rate.
Wu et al. (2022)	Cross Sectional	Multiple cities and states	Asian American sample in the USA (n=916 with complete cases)	N/A	Direct experiences of racial discrimination (9‐items), perceptions of cultural racism (4‐items), and anticipatory racism (4‐items)	Intention to purchase a gun since COVID (1‐item), purchased a gun since COVID (1‐item), intention to purchase ammunition since COVID (1‐item), purchased ammunition since COVID (1‐item), and firearm carriage since COVID (1‐item)	Racial discrimination (direct) was associated with gun purchasing, intention to purchase ammunition, and purchasing of ammunition during COVID‐19. Cultural racism was positively associated with gun purchasing. Among gun owners, direct racial discrimination was positively associated with the likelihood of carrying a gun more frequently during COVID‐19
Wu et al. (2025)	Cross sectional survey analysis	Multiple cities and states	916 self‐identified Asian American across the USA	Minority Stress Theory	Direct experiences of racial discrimination (9‐items), perceptions of cultural racism (4‐items), and response to racism (4‐items)	Firearm acquisition/purchase (1 item)	Analysis of indirect effects indicated that greater experiences of racism were associated with increased alcohol use, which was in turn linked to a higher likelihood of firearm purchase. Similarly, higher levels of mental distress were associated with greater alcohol use, which was also linked to a higher likelihood of purchasing a firearm. Experiences of racism were also related to higher mental distress, which was associated with greater alcohol use, and in turn, a higher likelihood of firearm purchase. However, the indirect pathway from racism to firearm purchase through mental distress alone was not statistically significant.
**Police Firearm Violence**							
Leslie et al. (2022)	CrossSectional	Census tracts	Census tracts (all of US)	N/A	Index of Dissimilarity; Interaction Index; Diversity Index		Residential racial segregation (index of dissimilarity) is associated with less fatal police shootings for Hispanic victims. Residential racial segregation (lower interaction index) is associated with more fatal police shootings for Black victims. Residential racial segregation (lower Diversity index) is associated with higher levels of fatal police shootings for Black and Asian victims.
Mesic et al. (2018)	Cross Sectional	States (minus RI)	United States (at State Level minus Rhode Island)	Threat hypothesis and Community Violence Hypothesis	Residential racial segregation (dissimilarity, isolation), Black‐White Disparity in (1) Incarceration Rate, (2) proportion of those without a college degree, (3) poverty rate, (4) median household income, (5)proportion of those who are renting as opposed to owning a home, (6) unemployment rate, and (7) adults not participating in the labor force ‐‐ aggregated & parsed into 5 components	Black‐White disparity in fatal police shootings	State racism index (overall measure of structural racism) is associated with a higher level of Black‐White disparity in fatal police shootings. Residential racial segregation is associated with a higher level of Black‐White disparity in fatal police shootings. Economic disparity is associated with a higher level of Black‐White disparity in fatal police shootings. Employment disparity is associated with a higher level of Black‐White disparity in fatal police shootings
Rafail (2024)	Retrospective correlational study	Multiple cities and states	National data from 2014‐2018 analyzed at the county‐level	N/A	Index of Dissimilarity	Fatal police shooting	There were significant racial disparities in fatal police shootings of youth based on race. The ratio of fatal police shootings to 100,000 youth ranged from 0.64 ‐ 0.86 for black youth, 0.27 ‐ 0.34 for Latinx youth, and 0.14 ‐ 0.22 for white youth. Specifically, residential racial segregation—as operationalized by ICErace – was indirectly associated with future youth firearm aggression by increasing neighborhood disadvantage and exposure to violence.
Siegel et al. (2019)	Cross Sectional	75 largest cities	Largest 69 cities in the US, fatal police shootings from 2013‐2017	Racial Threat Hypothesis	Index of Dissimilarity	Black‐White disparity in fatal police shootings	Residential racial segregation is associated with higher levels of Black‐White disparities in fatal police shooting rates
Siegel et al. (2021)	Cross Sectional	14,014 censustracts, 75 cities and 32 states	Part 1 (fatal shooting in census tract): 14014 Census Tracts, 75 Cities, and 32 States Part 2 (Black fatal shooting victims from police): 557 incidents were non‐hispanic Black, 125 1Census Tracts, 75 Cities, and 32 States	Social conflict hypothesis (minority threat hypothesis), Community violence hypothesis	Index of Dissimilarity (city‐ and state‐level); Black‐White disparity in adults without a college degree (state‐level)	Fatal Police shootings; Fatal Police Shootings of Black Victims Only	Black‐White disparity in educational attainment (i.e., adults ages 25 or higher) was positively associated with fatal shootings, while the index of dissimilarity (less residential segregation) was associated with more fatal shootings. For part 2 (analysis focused on Black victims of fatal police shootings), index of dissimilarity was positively associated with the fatal police shootings of Black victims.

## RACISM AND INTERPERSONAL FIREARM VIOLENCE

### Methodological qualities

#### Theoretical foundation

Of the 34 studies examining racism and interpersonal firearm violence, 16 studies did not mention any theoretical model or framework in introducing research questions, hypotheses, and interpreting results (see Table 1). The other 18 studies applied one or more theories to explain how structural racism contributes to negative structural and social processes (e.g., low social capital, neighborhood physical disorder) that may, in turn, lead to experiences with interpersonal firearm violence. A substantial number of studies drew on theories emphasizing structural determinants of health and violence, including Ecosocial Theory (5 studies; *theory described in* Krieger, 2012), Racial Stratification Theory (2 studies; *theory described in* Bonilla‐Silva, 1997; Stokely & Hamilton, 1967), Conflict Theory (2 studies; *theory described in* Petrocelli et al., 2003), Critical Race Theory (1 study; *theory described in* Delgado & Stefancic, 1998), Levels of Racism (1 study; *theory described i*n Jones, 2000), Socioecological Model (1 study; *theory described in* Bronfenbrenner, 1977), and Intersectionality Theory (1 study; *theory described in* Crenshaw, 1997). For instance, Ecosocial Theory was the most frequently cited theory and posits that health, disease burden, and well‐being within and across generations are largely influenced by “…the multilevel dynamic and co‐constituted societal and ecologic context within which we live, work, love, play, fight, ail, and die” (Krieger, 2016). Moreover, Benns et al. (2020) used the Levels of Racism model to support a multidimensional conceptualization of racism. These theories, in turn, theories emphasized the structural determinants underlying racism and racial disparities in firearm violence.

Theoretical models from criminology such as Social Disorganization Theory (3 studies; *theory described in* Sampson & Groves, 1989), Strain Theory (1 study; *theory described in* Agnew, 1985), Contact Hypothesis Theory (1 study; *theory described in* Allport, 1954; Pettigrew, 1998), Social Control Theory (1 study; *theory described in* Hirschi, 1998), and Code of the Street (1 study; *theory described in* Anderson, 1999) were also leveraged to explain how racism contributes to interpersonal firearm violence. For instance, Social Disorganization Theory was leveraged to theorize that residential racial segregation may contribute to firearm violence by breaking down community structures, weakening social bonds, and reducing opportunities for informal social control (e.g., Ousey & Augustine, 2001). These criminological theories, taken together, highlight processes such as neighborhood characteristics, weakened informal controls, and norms that enable or constrain violent behaviors.

Finally, in contrast to structural or criminological theories, which emphasize upstream systems or community processes that shape racial health disparities and crime, these theories focus on the individual‐level factors, explaining how personal experiences, exposures, and coping responses shape firearm‐related behaviors and outcomes across time. Studies drew on individual and life course perspectives, including Life Course Perspective (2 studies; Elder, 1998), Cumulative Disadvantage Theory (1 study; *theory described in* Sampson & Laub, 1997), Minority Stress Theory (1 study; *theory described in* Meyer, 2003), and the Coping Model of Protective Gun Ownership (1 study; *theory described in* Buttrick, 2020), highlighting how individual experiences, stress processes, and developmental trajectories shape firearm violence over time within the context of racism.

#### Racism measurement

The most commonly used measure of racism was residential racial segregation (16 studies; Massey & Denton 1998) and the Home Owners' Loan Corporation (HOLC) redlining data (13 studies; Greer 2013). Measures of residential racial segregation quantify the degree to which racial groups are spatially separated into different neighborhoods within a larger region (e.g., city, state; Knopov et al. 2019). While multiple residential racial segregation were used (e.g., isolation/entropy index; Siegel et al., 2023), the index of dissimilarity was the most common (used in 11 of the 16 studies), indicating the percentage a specific racial group would have to relocate to achieve an even distribution across geographic units in a broader area. Historical redlining data reflects a discriminatory mortgage lending practice where neighborhoods were categorized based on their perceived financial risk, often influenced by the racial composition of the residents (e.g., Jacoby et al. 2018). The HOLC ranked neighborhoods for lending using four categories: best (green), desirable (blue), declining (yellow), and hazardous (red), with the composition of yellow‐ and red‐lined areas primarily consisting of immigrants and racial minorities. In turn, neighborhoods—namely, census tracts (e.g., Poulson et al., 2023a, 2023b), block groups (e.g., Benns et al., 2020), and zip‐codes (e.g., Mehranbod et al., 2022) ‐ classified as “definitely declining” and “hazardous” were associated with higher levels of interpersonal firearm violence. Eight studies also examined racialized economic segregation using the index of concentration at the extremes (ICE), which measures the spatial polarization of low‐income Blacks to high‐income Whites across census tracts (e.g., Krieger et al., 2017). Of note, Uzzi et al. (2024) adapted ICE to spatially contrast non‐Hispanic, White individuals who received prime mortgages versus non‐Hispanic, Black individuals who received subprime loans. Black–White ratios were also computed in nine studies across socioeconomic outcomes (e.g., median income, unemployment rate, and incarceration rates) to assess racial inequities across multiple institutions, reflecting historical and ongoing discriminatory policies and practices that limit resources of Black communities (e.g., Wong et al., 2020). Lastly, in three studies, self‐report measures were used to assess multiple forms of racial discrimination, including interpersonal and cultural forms of racism‐related experiences (e.g., Wu et al., 2022). Finally, researchers also used novel measures of racism, including the murder of George Floyd as an inflection point in the temporal analysis of firearm violence (Larson et al., 2023), Black–White disparities in environmental hazards (Siegel et al., 2023b), and county‐level historical enslavement rates within the southeast region of the US (Buttrick & Mazen, 2022).

Nineteen studies incorporated multiple measures of racism (e.g., Siegel et al., 2023a). Siegel and colleagues, for instance, developed latent factor scores at the city‐ (Siegel et al., 2023a) and state‐level (Siegel et al., 2023b) using multiple indices of structural racism (e.g., Black–White disparities in socioeconomic outcomes).

#### Interpersonal firearm violence measurement

Thirteen studies measured interpersonal firearm violence by examining rates of firearm‐related homicides and non‐fatal firearm‐related assaults jointly (e.g., Schleimer et al., 2022). For instance, Benns et al. (2020) integrated data from a local police department, coroner's office, and level 1 trauma center to identify gunshot victims in Louisville, KY. Of note, one study, and the only one in our review, examined both fatal and nonfatal injuries, but only within the context of mass shooting (Ghio et al., 2023). Next, thirteen studies examined firearm homicides (e.g., Houghton et al., 2021), and six of these studies quantified Black–White disparities in firearm homicide victimization rate (e.g., Wong et al., 2020). Knopov et al. (2019), for example, computed a ratio of firearm homicide victimization rate between Black and White individuals across 42 states. Additionally, studies examining fatal firearm assault injuries primarily relied on open‐source, public health surveillance data (e.g., National Violent Death Reporting System), and analyses were conducted across multiple cities and states (e.g., Mehranbod et al., 2022). Using self‐report data, five studies investigated racism as a predictor of interpersonal firearm violence risk behaviors, including firearm carriage (e.g., Lee et al., 2025), possession (e.g., Buttrick & Mazen, 2022), and purchasing (e.g., Su et al., 2024). Finally, one study assessed firearm homicide offense rates for juveniles (ages 14‐17 years old; Ousey & Augustine, 2001), while another assessed youth firearm aggression (i.e., youth self‐report of using a firearm on someone; Lee et al., 2024).

Lastly, 26 studies analyzed a single interpersonal firearm outcome from one data source; four examined multiple outcomes (using a single data source), and three used multiple data sources (to study a single outcome). A study by Krieger et al. (2017), however, measured two outcomes (i.e., non‐fatal firearm assaults, fatal firearm assaults) using two sources of data (i.e., Massachusetts Department of Public Health vital statistics and Weapon‐Related Injury Surveillance System).

#### Data recency for outcome

Aggregated Measures of interpersonal firearm violence across multiple years were used in 31 of the 34 studies. To assess data recency, we used the earliest year of data for a conservative estimate. Of the 34 studies, only two studies measured interpersonal firearm violence less than 5 years ago (2021–2025) and 20 studies measured it 5 to 9 years ago (2011–2020).

#### Scope of sample

The scope of the sample was categorized into three levels: (1) city (10 studies; e.g., Benns et al., 2020; Poulson et al., 2023a, 2023b), (2) state (3 studies; e.g., Shour et al., 2023), and (3) national (21 studies; e.g., Wu et al., 2022). Studies at the city‐level (e.g., Philadelphia, Flint) assessed structural racism, interpersonal firearm violence, and relevant moderators and mediators at the census tract (e.g., Uzzi et al., 2022), block group (e.g., Benns et al., 2020), or block level within city limits (e.g., Poulson et al., 2021; Poulson et al., 2023a, 2023b), or at the individual level (e.g., Lee et al., 2024). At the state‐level, three studies examined the influence of structural racism (e.g., racialized economic segregation, residential racial segregation) on firearm assaults among census tracts (Krieger et al., 2017; Uzzi et al., 2024) or counties (Shour et al., 2023). Finally, among studies that analyzed data spanning multiple cities and states, four studies assessed racism and firearm violence at the state level (e.g., Conrick et al., 2023), seven studies at the city level (e.g., Gobaud et al., 2024), three studies at the zip code level (e.g., Spitzer et al., 2006), two studies at the census tract level (e.g., Siegel & Nicholson‐Robinson, 2025), three studies at the individual level (e.g., Wu et al., 2025), and one study used a grid of hexagons (roughly the size of a New York City block; Hans et al., 2025). Six studies also had nested data (e.g., zip codes nested in cities; Mehranbod et al., 2022). Finally, studies that examined multiple cities and states utilized qualifiers to include certain zip codes, cities, or states. For instance, one study focused on cities with populations exceeding 100,000 (e.g., Wong et al., 2020), while another study excluded states that reported fewer than 10 Black homicides over the past 25 years (e.g., North Dakota, Delaware; Knopov et al., 2019).

#### Analytic approach

Twenty‐two studies used cross‐sectional or time‐lagged cross‐sectional data (i.e., the association between redlining and firearm homicide rate; for example, Benns et al., 2020) to evaluate the association between racism and interpersonal firearm violence. Longitudinal analyses were used in 12 studies to evaluate whether racism contributed to changes in firearm violence outcomes over time. Schleimer et al. (2022), for instance, conducted a difference‐in‐differences analysis and observed that cities with a higher level of racialized economic segregation observed greater increases in fatal and non‐fatal firearm assault injuries from 2018/2019 to 2020. Mediators (5 studies; e.g., Poulson et al., 2021) and moderators (3 studies; e.g., Poulson et al., 2023a, 2023b; Uzzi et al., 2022) were also assessed in a few studies. For instance, Poulson et al. (2021) assessed neighborhood‐level socioeconomic conditions, housing‐related factors, and racial composition as mediators in the association between historical redlining and firearm shooting incidents. For moderators, Uzzi et al. (2023) used an additive interaction approach to test the synergistic effect of historical redlining and contemporary racialized economic segregation on non‐fatal shootings.

Lastly, while measures of racism and interpersonal firearm violence were geocoded in the vast majority of studies, only three controlled for spatial autocorrelation (e.g., Jacoby et al., 2018). Other studies used multilevel modeling or cluster‐robust standard errors to account for non‐independence (e.g., individuals nested in census tracts; e.g., Lee et al., 2025).

### Key findings for racism and interpersonal firearm violence

Key findings are organized based on the type of racism involved, including: (1) residential racial segregation, (2) redlining, (3) racialized economic segregation, (4) Black–White inequities in socioeconomic outcomes, (5) interpersonal and cultural racial discrimination, and (6) novel racism measures.

#### Residential racial segregation

Twelve of the 16 studies documented the association between residential racial segregation and firearm homicide (e.g., Houghton et al., 2021), non‐fatal firearm assault (e.g., Krieger et al., 2017), and residential racial segregation and Black–White disparities in firearm homicide (e.g., Siegel & Nicholson‐Robinson, 2025). Houghton et al. (2021), for instance, observed that a higher value on the index of dissimilarity was associated with higher firearm homicide. Of note, Krieger et al. (2017) did not observe an association between the index of dissimilarity and firearm assaults, positing that the index of concentration at the extremes (by race) can predict steeper gradients in fatal and non‐fatal firearm assaults than the index of dissimilarity when measuring residential segregation (Krieger et al., 2017). Further, Lee et al. (2024) observed an indirect effect of residential racial segregation, but not a direct effect on interpersonal firearm violence, suggesting that this relationship may be undergirded by downstream mechanisms (e.g., community violence).

#### Historical redlining

Eleven of the 12 studies examining redlining documented its association with interpersonal firearm violence (e.g., Dholakia et al., 2024; Uzzi et al., 2024). Neighborhoods classified as “hazardous” (i.e., redlined) demonstrated higher rates of firearm homicide (Mehranbod et al., 2022), non‐fatal firearm assaults (Uzzi et al., 2022), and a combination of non‐fatal firearm assault and homicide than neighborhoods classified as “best” (Benns et al., 2020). For instance, Jacoby et al. (2018) observed that census tracts in historically redlined areas had an eightfold increase in firearm violence incidents relative to green‐lined census tracts, and yellow‐lined census tracts showed a sevenfold increase in firearm violence. Moreover, three studies examined moderators (Poulson et al., 2023a, 2023b; Uzzi et al., 2022, 2024) and one study examined mediators (Poulson et al., 2021) in the association between redlining and interpersonal firearm violence. For mediators, neighborhood‐level socioeconomic indicators (i.e., a combination of poverty rate, limited educational attainment, and need for public services) mediated the association between historical redlining and firearm violence. With regard to moderators, when black income mobility reached $50,000 or higher, the disparity in firearm shooting rates between redlined (undesirable for investment) and green‐lined census blocks (desirable for investment) diminished (Poulson et al., 2023a, 2023b). Thus, socioeconomic conditions like income mobility, concentrated poverty, and racialized economic segregation may shape the lasting impact of historical redlining on present interpersonal firearm violence.

#### Racialized economic segregation

Seven of nine studies documented the influence of racialized economic segregation on: (1) non‐fatal firearm assault (e.g., Krieger et al., 2017), (2) firearm homicide (e.g., Krieger et al., 2017), (3) a combination of firearm homicide and non‐fatal assault (Schleimer et al., 2022), (4) Black–White disparities in the firearm homicide rate (e.g., Siegel et al., 2023b), and (5) the self‐reported frequency of youth firearm carriage (Lee et al., 2025). Schleimer et al. (2022), for instance, observed that zip codes with greater spatial polarization between low‐income Blacks and high‐income Whites predicted a steeper increase in fatal and non‐fatal firearm assault injuries. Uzzi et al. (2024) found the highest firearm violence in census tracts that were both historically red‐ or yellow‐lined and in the upper half of contemporary racialized economic segregation. Krieger et al. (2017) found that ICE by race and income was more predictive of firearm violence than the index of dissimilarity, noting it provides a more localized measure of structural racism. Krieger et al. (2017) found that ICE by race and income was more predictive of firearm violence than the index of dissimilarity, noting it provides a more localized measure of structural racism.

#### Black–White inequalities

Six of eight studies documented that Black–White inequalities in socioeconomic indices (e.g., poverty rate) and, in some studies, incarceration rate (Siegel et al., 2024), political participation (Siegel et al., 2023a), and environmental racism (Siegel et al., 2023b) were associated with Black–White disparities in homicide rates. Importantly, six studies summarized Black–White inequalities as a single score by computing averages or dimension reduction techniques (e.g., factor analysis) at the census tract or state level (Siegel et al., 2024). For instance, Siegel and colleagues (i.e., 2023a, 2024) developed measurement models for structural racism at the county and state level and correlated the latent factors to Black–White disparities in the firearm homicide rate.

#### Interpersonal and cultural racial discrimination

Three studies assessed interpersonal and cultural forms of racial discrimination and firearm violence risk. Two of those studies analyzed a national sample of Asian American adults living in the US during COVID‐19 (Wu et al., 2022, 2025), while one study examined self‐reported experiences of racism in a national sample of adults using the Health, Ethnicity, and Pandemic (HEAP) study. These studies, when taken together, unveiled that interpersonal forms of racism (e.g., Anti‐Asian racism, racial hate crimes) and exposure to cultural racism (e.g., negative portrayal of Asian Americans in the media) are associated with firearm violence risk behaviors (e.g., purchasing firearms, firearm carriage). Unlike most of the other studies discussed in this review, these three studies used self‐report surveys to assess firearm violence risk behaviors (e.g., firearm carriage), which is critical for informing individual‐level interventions.

#### Novel measures of racism

Three studies tested if novel measures of structural racism contribute to interpersonal firearm violence. Uzzi et al. (2024) computed census tract‐level ICE scores to approximate racialized subprime mortgage lending—that is, a concept related to racial capitalism—and found that it's associated with firearm assaults. Buttrick and Mazen (2022) assessed the historical enslavement rates across Southern counties (i.e., the proportion of the total populations that were enslaved from the 1860 Census) and found that it was directly associated with firearm possession, and indirectly associated via perceptions of safety using the Gallup Daily Tracking Poll aggregated at the county level. Finally, Larson et al. (2023) examined level and trend changes in firearm assault injuries following George Floyd's killing, and documented that this racism‐related incident contributed to a sevenfold increase in the 3 months after in Minneapolis, MN. These three studies found that these novel indicators of structural racism can influence interpersonal firearm violence.

## RACISM AND POLICE FIREARM VIOLENCE

### Methodological qualities

#### Theoretical foundation

Three of five studies examining the association between racism and police firearm violence used the racial threat hypothesis (also known as the minority threat or social conflict hypothesis) to explain racial disparities in fatal police shootings (*theory described in* Jacobs & O'Brien, 1998; Smith, 2004). Mesic et al. (2018), for instance, used the racial threat hypothesis to suggest that police officers perceive predominantly Black and other racially minoritized communities as inherently more threatening and, consequently, use a disproportionate level of force in segregated, Black communities. Two of three studies that referenced the racial threat hypothesis also referenced the community violence hypothesis as a competing theory (Mesic et al., 2018; Siegel et al., 2021), positing that police firearm violence is a result of more crime and police‐civilian interaction in a particular neighborhood. Researchers suggested that a link between residential segregation and fatal police shootings would support the racial threat hypothesis, while an association between crime rates and fatal police shootings would support the community violence hypothesis (e.g., Mesic et al., 2018).

#### Racism measurement

All five studies assessed residential racial segregation using the index of dissimilarity (Leslie et al., 2022; Mesic et al., 2018; Siegel et al., 2019, 2021). Residential racial segregation was assessed using three measures: interaction index (i.e., the extent to which members of one racial group are exposed to members of another racial group within a geographic area), diversity index (i.e., racial diversity within a geographic area), and isolation index (i.e., the extent to which members of one racial group are only exposed to members of their own group; see Massey & Denton, 1998). Three studies also examined Black–White inequalities in socioeconomic status and incarceration rate (Mesic et al., 2018; Siegel et al., 2021). Mesic et al. (2018), for instance, developed a measure of statewide structural racism using five composite scores: (1) residential segregation by aggregating the index of dissimilarity and the isolation index; (2) educational disparity, measured by the Black–White gap in the proportion of those without a college degree; (3) economic disparity, assessed through the Black–White gap in the proportion of people living below the poverty level, the difference in median income between Black and White populations, and the disparity in the proportion of individuals living in rental properties versus homeownership; (4) incarceration disparity, defined by the Black–White incarceration rate gap; and (5) employment disparity, analyzed using the Black–White gap in the proportion of people not participating in the labor force and the disparity in unemployment rates. Lastly, contrary to studies examining interpersonal firearm violence, studies of police firearm violence did not examine historical redlining, as well as interpersonal or cultural racism.

#### Police firearm violence measurement

Three of the four studies utilized the database developed by the Mapping Police Violence (MPV; Sinyangwe et al., 2023) project (e.g., Siegel et al., 2021). MPV data captures incidents published in the news in which a law enforcement officer, whether off‐duty or on‐duty, used lethal force on a civilian that resulted in the civilian's death (see Sinyangwe et al., 2023 for more detail). Data construction entails screening news report of police violence; recording civilians' and officers' demographics and incident details; cross‐validating incidents with external data sources (e.g., Washington Post's Fatal Force Data); and geocoding incidents. Mesic et al. (2018) used MPV data to construct Black–White disparities in fatal police shootings across states, while Siegel et al. (2021) used MPV data to calculate the number of Black and White fatal police shooting victims separately. Leslie et al. (2022) utilized the Washington Post's Fatal Force Database (FFD)—a validated, crowdsourced dataset on fatal police shootings similar to MPV (Comer & Ingram, 2023; Leslie et al., 2022). Moreover, Rafail (2024) integrated data from four major databases—that is, Fatal Encounters, The Washington Post's Fatal Force, The Guardian's The Counted, and the U.S. Police‐Shooting Database—to create a merged dataset cross‐referenced across multiple sources. Finally, with regards to the number of data sources used, two studies assessed multiple police firearm violence outcomes (Leslie et al., 2022; Siegel et al., 2021), such as Asian, Hispanic, and Black fatal police shooting rates, separately.

#### Data recency for outcome

Police firearm violence rates were aggregated over the course of multiple years, with the earliest year to provide a conservative estimate of data recency. Four studies aggregated police firearm violence measures that were 10+ years ago, and one study aggregated data from 5 to 9 years ago.

#### Scope of sample

The sample across all five studies spanned multiple cities and states, which strengthens the generalizability of the study findings, particularly to US urban areas.

#### Analytic approach

All five studies utilized cross‐sectional data and used regression‐based approaches (e.g., ordinary least squares, generalized linear models) to approximate associations between racism and police firearm violence. Two studies estimated multilevel models to evaluate whether census tract‐, city‐, and state‐level covariates (e.g., index of dissimilarity) were associated with fatal police shooting incidents at the census tract level (Rafail, 2024; Siegel et al., 2021).

### Key findings for racism and police firearm violence

Key findings are organized based on the specific type of racism involved, including: (1) residential racial segregation and (2) Black–White inequalities in socioeconomic status and incarceration rates.

#### Residential racial segregation

All five studies found that residential racial segregation was associated with a higher rate of fatal police shooting incidents in a census tract (e.g., Siegel et al., 2021), larger Black–White disparities in fatal police shootings (e.g., Mesic et al., 2018), and higher fatal police shooting rates for Black, Hispanic, and Asian victims (e.g., Leslie et al., 2022). For instance, Leslie et al. (2022) found that census tracts with a higher concentration of racial minority members (i.e., diversity index) and fewer interactions between racial minority groups due to segregation (i.e., interaction index) were more likely to have fatal police shootings involving Black victims. This finding suggests that racially segregated communities are more vulnerable to fatal police shootings.

#### Black–White inequalities in SES

Black–White inequalities in socioeconomic indices were associated with larger Black–White disparities in fatal police shooting rates (e.g., Mesic et al., 2018) and a higher likelihood of a fatal firearm assaults (e.g., Siegel et al., 2021). Further, Mesic et al. (2018) found that Black–White inequities in economic (i.e., poverty rate, median household income, rate of households renting) and employment outcomes (i.e., unemployment rate, proportion of people not participating in the labor force) were associated with a larger Black–White disparity in fatal police shootings of unarmed victims, which is an indicator of racial disparity in police firearm violence.

## DISCUSSION

The objective of this scoping review is to (1) summarize the quality of evidence, (2) identify the key findings in the extant body of literature, and (3) generate a series of future research priorities for the study of racism and firearm violence.

### Quality of evidence

The first objective of this scoping review was to evaluate the methodological approaches used in the study of racism and firearm violence, including theoretical application, measurement of racism and firearm violence, analytic approaches, and generalizability of study findings. The application of theory can help scholars in the field evaluate new hypotheses or validate existing ones about how racism influences racial disparities in firearm violence. Twenty‐one studies discussed one or more theoretical models from multiple disciplines to elucidate the social, structural, and intrapersonal processes underlying the connection between racism and firearm violence. The ecosocial theory, the most cited, suggests that racial disparities in firearm violence are shaped by factors that exist across multiple levels, including racism (e.g., redlining) and its downstream consequences (e.g., community violence; Krieger, 2016). Most of the employed models, however, do not offer racism‐related mechanisms that shape racial disparities in firearm violence. For instance, while the social disorganization theory emphasizes the community's ability to maintain effective social controls to prevent firearm violence, these models do not elaborate on how racism shapes social disorganization. A theoretical model that elucidates the multilevel pathways linking different types of racism with firearm violence is needed to help identify intervention points for addressing racial disparities in firearm violence.

Next, in examining the measurement of racism, the vast majority of studies leveraged an administrative and open‐source data, such as the American Community Survey (ACS) and geocoded HOLC maps (Aaronson et al., 2021). For instance, residential racial segregation indices (e.g., dissimilarity, isolation index), racialized economic segregation, and racial inequalities in socioeconomic outcomes were calculated using ACS data. While these measures can assess structural racism over a geographic area, life experiences of interpersonal, institutional, cultural, and structural racism may be highly variable and nuanced. Two racially minoritized members living in the same census tract may have different lived experiences with racism. Moreover, relying solely on structural racism measures might overlook other manifestations of racism, which may also contribute to firearm violence. For instance, news media have often engaged in episodic reporting (i.e., focusing on individual incidents) without applying a public health framing. This, in turn, may reinforce racial prejudice and discriminatory policing practices and exacerbate racial disparities in police firearm violence. Of note, one study investigated the effect of racist incidents on interpersonal firearm violence (e.g., examining the killing of George Floyd; Larson et al., 2023), while others constructed statistical measurement models of structural racism using factor analytic methods (e.g., state structural racism index; Siegel et al., 2024).

With regards to measures of firearm violence, all but five studies utilized public health surveillance data (e.g., National Violent Death Reporting System) or databases that log real‐time media accounts of fatal police shootings (e.g., MPV). These epidemiologic and open‐source databases offer insights into the rate of firearm violence at different geographic levels (e.g., census tracts, states). Moreover, while databases like MPV provide incident‐level information about fatal police shootings (e.g., race/gender of victim, whether victim was armed), this data is not without limitations. First, most of the interpersonal firearm violence measures assess victimization but not perpetration, with the exception of the FBI Supplementary Homicide Report. Lee and colleagues (2025) also examined the indirect influence of racialized economic segregation on youth firearm aggression (using a firearm on someone) using the Flint Youth Injury study (Cunningham et al., 2015). Understanding factors underlying perpetration can help identify risk factors associated with firearm violence involvement. Second, firearm violence surveillance data do not assess individual‐level firearm injury risk behaviors such as firearm carriage or illegal firearm purchasing. Of note, four of the five studies that used surveys measured risk factors proximal to firearm violence, such as firearm carriage (e.g., Wu et al., 2025). Third, surveillance data do not capture the situational context in which the firearm violence incident occurs. For example, firearm homicide could be a result of interpersonal conflict, intimate partner violence, or burglary. Understanding the situational context of firearm violence incidents can inform prevention strategies specific for different types of firearm violence. Finally, while most studies combined firearm violence data for extended periods (e.g., over a decade), examining temporal trends can provide insights into how racism shapes the trajectory of firearm violence over time (see both Larson et al., 2023 and Wong et al., 2020).

Further, 27 studies assessed firearm violence across geographic areas spanning multiple cities and states, while the remaining 12 studies examined firearm violence within a single city or state. While the external validity of findings increases with a more representative sample (i.e., multiple states and cities), there were some limitations to sampling approaches. First, excluding cities based on their population size (e.g., <100,000) may restrict our ability to generalize findings to smaller urban areas also adversely impacted by structural racism and firearm violence. It's conceptually reasonable that large cities like Boston, for instance, may have greater societal resources (e.g., more job opportunities), which may attenuate the influence of racism on firearm violence. Second, research is needed to assess additional contextual factors (e.g., gun policies, local government spending) to better understand how structural racism influences firearm violence across different urban areas. For instance, residents in racially segregated urban areas may encounter lower rates of firearm violence due to the type of firearm safety policies implemented at the state‐level. Finally, racism may also impact firearm violence within racial and ethnic minority groups (Centers for Disease Control and Prevention, 2021), as well as those living at the intersections of racial and gender minoritization (e.g., Black trans women; Wirtz et al., 2020).

Lastly, from an analytic perspective, several strengths emerged. Eight studies used longitudinal data to assess whether structural racism influenced interpersonal firearm violence over time. A few studies employed mediation analyses to examine pathways from racism to violence, and three tested moderators, though only one focused on protective factors. However, notable limitations were also observed. First, only four studies using localized place‐based data (e.g., census tracts) accounted for spatial dependencies. This is a limitation as rates of firearm violence in one area can influence rates in nearby areas, and, if not accounted for, can lead to misleading conclusions. Second, while multiple indicators of structural racism were included in several studies, these indicators may be strongly, positively correlated, contributing to multicollinearity issues. Many place‐based measures of structural racism were also sourced from the same data using similar variables (e.g., ACS), potentially exacerbating multicollinearity issues. Third, spatial misalignment was observed between the measurement of firearm violence (zip code level) and racism (census tract level). Drawing conclusions about zip codes using census tract data can limit the ecological validity of findings. Lastly, because of limited data on firearm‐related outcomes beyond fatal and nonfatal injuries, some researchers used epidemiologic indicators (e.g., the percentage of suicides involving a firearm) as proxies for county‐level gun ownership.

### Synthesis of findings

The second objective of this scoping review was to synthesize evidence regarding the influence of racism on racial disparities in interpersonal and police firearm violence. Emerging evidence suggests that structural, interpersonal, and cultural racism may contribute to racial disparities in both interpersonal and police firearm violence. Measures of racism utilized in studies varied, including measures of structural racism (e.g., residential racial segregation), legacies of racially discriminatory policies (e.g., historical redlining, historical rates of enslavement), racist incidents (killing of George Floyd), and self‐reported interpersonal and cultural racism. Residential racial segregation, the most commonly assessed indicator of structural racism (21 of the 39 studies), was associated with higher levels of Black–White disparities in firearm homicide and fatal police shootings. Racial segregation may not only prevent racially minoritized communities from accessing key economic and societal resources but also foster deleterious social conditions, such as community violence, which increases the likelihood of firearm violence. Finally, one study found that neighborhood disadvantage and community violence mediated the link between racial segregation and youth firearm aggression. Consistent with the FSRH (Williams & Mohammed, 2013), the implication of these findings is that ameliorating the structural consequences of residential racial segregation may be critical to reducing racial disparities in firearm violence. For instance, initiatives that promote upward socioeconomic mobility for racially minoritized youth and adults may help mitigate c firearm violence (Poulson et al., 2023a, 2023b). Such efforts may include creating opportunities for workforce training and accessing higher education in racially segregated communities (Kearney & Levine, 2014).

Concerning police firearm violence, the police may perceive racially segregated communities as inherently threatening, leading to over‐policing and greater police shootings in these communities (Siegel et al., 2021). Reducing racial disparities in fatal police shootings could involve structural interventions, such as community‐led governance approach may be promising strategies for promoting fair and just policing practices (Hodgkinson et al., 2019). Further, racist practices are deeply entrenched in modern‐day policing through tactics like stop‐and‐frisk. Researchers have contended that police departments should cut their ties to their long‐held racist practices by investing in restorative justice practices, community well‐being programs, and crime prevention instead of mass incarceration (Brewer & Heitzeg, 2008). For instance, investing in the structural determinants of safety (e.g., greening vacant lots and supporting neighborhood revitalization efforts) and violence interrupters instead of focusing on punitive tactics alone may help reduce racial disparities in police firearm violence.

Next, associations between historical redlining and interpersonal firearm violence have been documented in thirteen studies (e.g., Jacoby et al., 2018). While the Fair Housing Act of 1968 legally abolished the practice of redlining, historically redlined neighborhoods continue to face economic challenges, disinvestment, and racial segregation (Poulson et al., 2021). Redlined neighborhoods consistently exhibited higher rates of firearm assaults compared to neighborhoods that were historically designated as favorable for investment (i.e., blue‐ and green‐lined neighborhoods). It is critical that policies and practices address the racially segregated structure of American cities, a legacy reinforced by historical practices such as redlining. Inclusionary zoning practices, funding the production and preservation of affordable housing, and low‐income housing tax credit are policies that may counteract the long‐term effects of redlining. Of note, five studies assessed moderators and mediators in the link between racism and firearm violence. Concerning moderators, opportunities for upward income mobility for Black Americans (Poulson et al., 2023a, 2023b), reducing racialized economic segregation (Uzzi et al., 2022), and reducing racialized subprime mortgage (Uzzi et al., 2024) lending could ameliorate the influence of historical redlining on interpersonal firearm violence. These studies highlight that structural interventions that promote social and economic opportunities could mitigate the occurrence of firearm violence in economically disinvested, racially minoritized communities. With regards to mediators, redlining predicted firearm incidents through neighborhood socioeconomic disadvantage (Poulson et al., 2021).

Racialized economic segregation is a measure that evaluates racial inequalities in economic deprivation and privilege (Krieger et al., 2017). Seven of nine studies documented a positive association between racialized economic segregation and interpersonal firearm violence (e.g., Schleimer et al., 2022). Moreover, community violence mediated the influence of racialized economic segregation on youth firearm carriage—a near‐term risk factor for youth firearm violence (Lee et al., 2025). Per the FSRH, racially minoritized members in communities with high levels of racialized economic segregation contend with higher levels of disinvestment and socioeconomic challenges, leading to concentrations of crime, urban decay, and abandoned properties in racially minoritized neighborhoods (Williams & Mohammed, 2013). Therefore, these studies highlight the importance of tackling economic deprivation within racially segregated neighborhoods. To reduce racialized economic segregation and prevent firearm violence, strategies such as community development initiatives that support local entrepreneurs, investment in job training and placement programs, and the development of affordable and mixed‐income housing are essential.

Racial inequalities (often Black–White inequalities) in socioeconomic outcomes and incarceration rates were also assessed as determinants of interpersonal and police firearm violence across 11 studies (e.g., Mesic et al., 2018). Black–White inequalities in socioeconomic and incarceration outcomes reflect the consequences of the enduring and persistent legacy of structural racism. For example, racial inequalities in socioeconomic and criminal justice outcomes may be a by‐product of racially discriminatory housing practices (e.g., redlining, racial covenants), which, in separate studies, are linked to negative police‐community interactions and community violence. These measures signal the need for institutionalized and sustainable policies to promote racial equity along multiple sectors of society (e.g., criminal justice system, labor market) to prevent firearm violence. Specifically, interventions should seek to eliminate or redesign policies and programs that disproportionately harm the health and socioeconomic well‐being of Black and other racially minoritized groups. Exclusionary zoning laws, for instance, have been shown to exclude Black residents from affordable housing and reinforce residential racial segregation (Zeimer, 2020), which, in separate studies, is linked with higher rates of police (Siegel et al., 2019) and interpersonal firearm violence (Knopov et al., 2019). Thus, interventions targeting structural practices and programs are critical to addressing racial disparities in firearm violence.

Self‐report assessments of interpersonal and cultural forms of racism were associated with firearm injury risk behaviors in three studies (e.g., firearm carriage; Wu et al., 2022). Two studies found that pandemic‐related anti‐Asian violence heightened fear for personal and family safety, increasing firearm purchasing for protection, with alcohol use mediating this link as a maladaptive coping response to interpersonal racism. Moreover, in a national study of US adults, self‐reported hate crime experiences were linked to firearm purchasing during the COVID‐19 pandemic. Therefore, researchers should consider how lived experiences of racism, in conjunction with place‐based exposures, influence firearm violence risk. Developing this research area, though currently limited to three studies, is necessary to comprehend how racism, in totality, influences racial disparities in firearm violence.

### Recommendations for future research

The final objective of this scoping review was to provide recommendations for future research based on the studies reviewed. Rather than reiterating the methodological or conceptual limitations already noted, we posit five recommendations for informing a second generation of research on racism and firearm violence research.

#### Transdisciplinary theoretical model

As noted in our review, 21 studies used theoretical models from multiple disciplines (i.e., public health, criminology, sociology, and psychology) to conceptualize how racism may contribute to interpersonal and police firearm violence. While these theoretical models posit specific, tenable pathways (e.g., social disorganization, community disinvestment), a transdisciplinary theoretical model can elucidate the multifaceted ways by which racism reinforces racial disparities in firearm violence. Guided by the National Institute on Minority Health and Health Disparities research framework, a transdisciplinary theoretical model posits that the determinants of firearm violence exist across multiple levels and domains (Alvidrez et al., 2019). For instance, racial disparities in police firearm violence may also be shaped by an interplay of community factors (e.g., lack of socioeconomic resources), cultural norms (e.g., media stereotyping predominantly Black, urban communities as dangerous), and socioenvironmental characteristics (e.g., community violence). Thus, a transdisciplinary model of racism and firearm violence can guide researchers in evaluating key pathways, and it can be refined with new findings to provide a foundation for future research.

#### Evaluate mediators

Only five studies assessed mediators (i.e., indirect effects) in the association between racism and interpersonal firearm violence, while the remaining 34 studies assessed direct effects. There are two noteworthy benefits of evaluating mediators. First, mediators play a distinct role for hypothesizing and empirically testing the theorized mechanisms by which racism affects firearm violence. Mediators elucidate key pathways and refine our understanding of how (not just whether) racism contributes to interpersonal and police firearm violence. The diversity of theoretical models leveraged across the studies points to a wide array of psychological, social, and structural processes, and thus, we recommend testing mediators. Identifying mediators can also help identify intervention areas to counteract the downstream negative effects of racism on firearm violence. For instance, the socioeconomic condition of a neighborhood mediated the association between redlining and the number of shooting incidents (Poulson et al., 2021). This finding suggests that the long‐term influence of racist housing practices on firearm violence can be mitigated, in part, by improving the socioeconomic outcomes of individuals living in racially segregated neighborhoods. As posited by the FSRH (Williams & Mohammed, 2013), addressing interpersonal firearm violence in racially segregated, urban areas should focus on addressing the socioeconomic disadvantages conferred by redlining. Thus, research is needed to identify key pathways from racism to firearm violence to inform equitable prevention approaches.

#### Identify protective factors

Only a single study assessed a protective factor, which plays a critical role in attenuating the deleterious effects of racism on firearm violence. Per resilience theory, socioecological resources (e.g., access to employment) and intrapersonal assets (e.g., future aspirations) may mitigate the adverse effects of racism on firearm violence and promote resilience among youth who develop in communities negatively shaped by structural racism (Zimmerman, 2013). For instance, the association between redlining and firearm shooting incidents was attenuated in neighborhoods where Black income mobility was $50,000 or higher (Poulson, Neufeld, LaRaja, et al., 2023a). This finding suggests that creating opportunities for social mobility in racially and economically segregated communities (e.g., employment opportunities, reparative actions) may mitigate the link between redlining and firearm violence. Additional research is needed to identify protective factors that span multiple levels.

#### Developmental perspective

Of the 39 studies, three examined the link between racism and youth interpersonal firearm violence, and none examined police firearm violence involving youth. Adopting a developmental perspective promotes firearm violence prevention programs that address the downstream consequences of racism at each life stage. This is especially important because firearm homicide rates are not static across the lifespan—that is, they rise sharply during adolescence and peak in emerging adulthood (ages 14–29), particularly among Black and Hispanic youth (Centers for Disease Control and Prevention, 2025). Researchers have theorized that different types of racism experiences (e.g., microaggressions) may affect health outcomes, such as firearm violence, differently across developmental periods (Gee et al., 2019). Thus, there is a need to examine how racism shapes interpersonal and police firearm violence during different developmental periods. For police firearm violence, societal biases often unjustly depict young Black males as criminals and threatening (Barbarin et al., 2020). Police officers, influenced by these biases, may perceive Black youth as less innocent, which may reinforce racial disparities in fatal police shootings among youth.

Moreover, applying a developmental perspective emphasizes the interplay of biological, psychological, social, and ecological factors across an individual's lifespan. Answering these research questions requires longitudinal data. By using longitudinal data, researchers can assess the cumulative effects and timing of racism experiences on firearm violence. Longitudinal studies can also highlight how experiences of racism and its downstream consequences shape firearm violence differently across life stages.

#### Improved firearm violence surveillance

The vast majority of studies (34 of 37 studies) used epidemiological surveillance data and administrative data (e.g., death certificates, police arrest records, and news articles) that reflect the fatal or nonfatal firearm assault injury enacted by other people or by the police. However, most data sources provide limited context about the shooting incident. Thus, there is a need for developing more comprehensive surveillance systems to measure firearm violence more accurately and to understand these incidents at a deeper level. For instance, most surveillance data do not measure multiple forms of interpersonal firearm violence, such as being threatened with a firearm, or specific sub‐types of firearm violence (e.g., intimate partner violence). We acknowledge that there are significant challenges in acquiring comprehensive data, given privacy concerns, and a lack of uniformity across data sources in how and what firearm violence data are collected. Akin to the MPV database, leveraging local, state, and national news sources could provide access to real‐time news media accounts on interpersonal firearm violence incidents and the related situational characteristics. Additionally, the use of national or statewide surveys to capture firearm violence risk behaviors (e.g., firearm theft) is sorely needed. Finally, better event data (e.g., body cam footage recording police firearm violence) is needed to understand the situational characteristics of police shootings.

### Limitations

Several limitations should be acknowledged. First, our review exclusively focuses on literature published in journals, and the exclusion of non‐English or gray literature could have led to the omission of relevant studies. Furthermore, it's possible that our search strategy may not have captured all potentially eligible studies. That said, we used a thorough method suitable for scoping reviews as described by Arksey & O'Malley (2005) and searched seven databases and checked references from key review papers. Moreover, we adapted Zaza's Guide to Community Preventive Services: Systematic Reviews and Evidence‐Based Methods scoring tool to evaluate the methodological quality of the reviewed studies (Zaza et al., 2000). It's not practical to use a randomized or non‐randomized control trial when studying the influence of redlining or residential segregation on firearm violence. Our reviewers evaluated each study based on five domains: (1) theoretical model, (2) measurement quality, (3) data recency, (4) analytic approach, and (5) generalizability.

## CONCLUSION

Our scoping review lays the groundwork for future research dedicated to assessing how racism contributes to the racial disparities in firearm violence. Our quality assessment highlights the need for a transdisciplinary theoretical model that elucidates the socioecological pathways linking racism to racial disparities in interpersonal and police‐related firearm violence. The transdisciplinary framework should be informed using community‐based participatory research practices to amplify the voices of communities most impacted by racism and firearm violence. Evaluating mechanisms from racism to firearm violence is also critical to advancing our understanding of how racism contributes to racial disparities in firearm violence. Utilizing both place‐based data and longitudinal survey data is essential for testing the multifaceted dynamics at play. Our review also emphasizes the importance of uncovering protective factors across different socioecological levels. Such insights could inform firearm violence prevention strategies that target the key pathways from racism to firearm violence. Finally, applying a developmental lens can illuminate the pivotal mechanisms at play during different development periods and offer insights into why racial disparities in firearm violence intensity during adolescence and endure into adulthood.

## CONFLICT OF INTEREST STATEMENT

The authors declare no conflicts of interest.

## Supporting information

Supporting File 1

Supporting File 2

## References

[ajcp70064-bib-0001] Covidence Systematic Review Software. (n.d.) [Computer software]. Veritas Health Innovation. www.covidence.org

[ajcp70064-bib-0002] Aaronson, D. , Faber, J. , Hartley, D. , Mazumder, B. , & Sharkey, P. (2021). The long‐run effects of the 1930s HOLC “redlining” maps on place‐based measures of economic opportunity and socioeconomic success. Regional Science and Urban Economics, 86, 103622. 10.1016/j.regsciurbeco.2020.103622

[ajcp70064-bib-0003] Agnew, R. (1985). A revised strain theory of delinquency*. Social Forces, 64(1), 151–167. 10.1093/sf/64.1.151

[ajcp70064-bib-0004] Allport, G. (1954). The nature of prejudice. Perseus Books.

[ajcp70064-bib-0005] Alvidrez, J. , Castille, D. , Laude‐Sharp, M. , Rosario, A. , & Tabor, D. (2019). The national institute on minority health and health disparities research framework. American Journal of Public Health, 109(S1), S16–S20. 10.2105/AJPH.2018.304883 30699025 PMC6356129

[ajcp70064-bib-0006] Anderson, E. (1999). Code of the street: Decency, violence, and the moral life of the inner city. W. W. Norton and Company.

[ajcp70064-bib-0007] Bailey, Z. D. , Krieger, N. , Agénor, M. , Graves, J. , Linos, N. , & Bassett, M. T. (2017). Structural racism and health inequities in the USA: Evidence and interventions. The Lancet, 389(10077), 1453–1463. 10.1016/S0140-6736(17)30569-X 28402827

[ajcp70064-bib-0008] Bancalari, P. , Sommer, M. , & Rajan, S. (2022). Youth exposure to endemic community gun violence: A systematic review. Adolescent Research Review, 7(3), 383–417. 10.1007/s40894-022-00178-5

[ajcp70064-bib-0009] Barbarin, O. A. , Tolan, P. H. , Gaylord‐Harden, N. , & Murry, V. (2020). Promoting social justice for African‐American boys and young men through research and intervention: A challenge for developmental science. Applied Developmental Science, 24(3), 196–207. 10.1080/10888691.2019.1702880

[ajcp70064-bib-0010] Benns, M. , Ruther, M. , Nash, N. , Bozeman, M. , Harbrecht, B. , & Miller, K. (2020). The impact of historical racism on modern gun violence: Redlining in the city of Louisville, KY. Injury, 51(10), 2192–2198. 10.1016/j.injury.2020.06.042 32650980

[ajcp70064-bib-0011] Bonilla‐Silva, E. (1997). Rethinking racism: Toward a structural interpretation. American Sociological Review, 62(3), 465–480. 10.2307/2657316

[ajcp70064-bib-0013] Brewer, R. M. , & Heitzeg, N. A. (2008). The racialization of crime and punishment: Criminal justice, color‐blind racism, and the political economy of the prison industrial complex. American Behavioral Scientist, 51(5), 625–644. 10.1177/0002764207307745

[ajcp70064-bib-0014] Bronfenbrenner, U. (1977). Toward an experimental ecology of human development. American Psychologist, 32(7), 513–531. 10.1037/0003-066X.32.7.513

[ajcp70064-bib-0015] Buttrick, N. (2020). Protective gun ownership as a coping mechanism. Perspectives on Psychological Science, 15(4), 835–855. 10.1177/1745691619898847 32375009

[ajcp70064-bib-0016] Buttrick, N. , & Mazen, J. (2022). Historical prevalence of slavery predicts contemporary American gun ownership. PNAS Nexus, 1(3), pgac117. 10.1093/pnasnexus/pgac117 36741447 PMC9896914

[ajcp70064-bib-0017] Carter, P. M. , & Cunningham, R. M. (2016). Adequate funding for injury prevention research is the next critical step to reduce morbidity and mortality from firearm injuries. Academic Emergency Medicine, 23(8), 952–955. 10.1111/acem.12982 27062328 PMC7182090

[ajcp70064-bib-0018] Centers for Disease Control and Prevention (2021). Web‐based Injury Statistics Query and Reporting System (WISQARS): Fatal and Non‐Fatal Injury Data. Centers for Disease Control and Prevention, National Center for Injury Prevention and Control. www.cdc.gov/injury/wisqars

[ajcp70064-bib-0019] Centers for Disease Control and Prevention (2025). WISQARS (Web‐based Injury Statistics Query and Reporting System). https://www.cdc.gov/injury/wisqars/index.html

[ajcp70064-bib-0020] Clark, R. , Anderson, N. B. , Clark, V. R. , & Williams, D. R. (1999). Racism as a stressor for African Americans: A biopsychosocial model. American Psychologist, 54(10), 805–816. 10.1037/0003-066X.54.10.805 10540593

[ajcp70064-bib-0021] Comer, B. P. , & Ingram, J. R. (2023). Comparing fatal encounters, mapping police violence, and Washington Post fatal police shooting data from 2015–2019: A research note. Criminal Justice Review, 48(2), 249–261. 10.1177/07340168211071014

[ajcp70064-bib-0022] Conrick, K. M. , Adhia, A. , Ellyson, A. , Haviland, M. J. , Lyons, V. H. , Mills, B. , & Rowhani‐Rahbar, A. (2023). Race, structural racism and racial disparities in firearm homicide victimisation. Injury Prevention, 29(4), 290–295. 10.1136/ip-2022-044788 36564165

[ajcp70064-bib-0023] Cooper, H. L. F. , West, B. , Linton, S. , Hunter‐Jones, J. , Zlotorzynska, M. , Stall, R. , Wolfe, M. E. , Williams, L. , Hall, H. I. , Cleland, C. , Tempalski, B. , & Friedman, S. R. (2016). Contextual predictors of injection drug use among black adolescents and adults in US metropolitan areas, 1993–2007. American Journal of Public Health, 106(3), 517–526. 10.2105/AJPH.2015.302911 26691126 PMC4815709

[ajcp70064-bib-0024] Crenshaw, K. (1997). Demarginalizing the Intersection of race and sex: A Black feminist critique of antidiscrimination doctrine, feminist theory and antiracist politics, feminist legal theories. Routledge.

[ajcp70064-bib-0025] Cunningham, R. M. , Carter, P. M. , Ranney, M. , Zimmerman, M. A. , Blow, F. C. , Booth, B. M. , Goldstick, J. , & Walton, M. A. (2015). Violent reinjury and mortality among youth seeking emergency department care for Assault‐Related injury: A 2‐year prospective cohort study. JAMA Pediatrics, 169(1), 63–70. 10.1001/jamapediatrics.2014.1900 25365147 PMC4306452

[ajcp70064-bib-0027] Delgado, R. , & Stefancic, J. (1998). Critical race theory: Past, present, and future. Current Legal Problems, 51(1), 467–491. 10.1093/clp/51.1.467

[ajcp70064-bib-0028] Dholakia, A. , Burdick, K. J. , Kreatsoulas, C. , Monuteaux, M. C. , Tsai, J. , Subramanian, S. V. , & Fleegler, E. W. (2024). Historical redlining and present‐day nonsuicide firearm fatalities. Annals of Internal Medicine, 177(5), 592–597. 10.7326/M23-2496 38648643

[ajcp70064-bib-0029] DiAquoi, R. C. (2018). Critical race life course perspective theory: A framework for understanding racism over the life course. International Journal of Qualitative Studies in Education, 31(1), 36–54. 10.1080/09518398.2017.1379622

[ajcp70064-bib-0030] Elder Jr. G. H. (1998). The life course as developmental theory. Child Development, 69(1), 1–12. 10.1111/j.1467-8624.1998.tb06128.x 9499552

[ajcp70064-bib-0031] Gee, G. C. , Hing, A. , Mohammed, S. , Tabor, D. C. , & Williams, D. R. (2019). Racism and the life course: Taking time seriously. American Journal of Public Health, 109(S1), S43–S47. 10.2105/AJPH.2018.304766 30699016 PMC6356137

[ajcp70064-bib-0033] Ghio, M. , Simpson, J. T. , Ali, A. , Fleckman, J. M. , Theall, K. P. , Constans, J. I. , Tatum, D. , McGrew, P. R. , Duchesne, J. , & Taghavi, S. (2023). Association between markers of structural racism and mass shooting events in major US cities. JAMA Surgery, 158(10), 1032–1039. 10.1001/jamasurg.2023.2846 37466952 PMC10357360

[ajcp70064-bib-0034] Gobaud, A. N. , Morrison, C. N. , Branas, C. C. , Jacoby, S. , Kramer, M. , & Adkins‐Jackson, P. B. (2024). Measuring the effect of historical structural racism on community firearm violence in US cities. Social Science & Medicine (1982), 361, 117355. 10.1016/j.socscimed.2024.117355 39321665 PMC11534521

[ajcp70064-bib-0036] Greer, J. (2013). The home owners’ loan corporation and the development of the residential security maps. Journal of Urban History, 39(2), 275–296. 10.1177/0096144212436724

[ajcp70064-bib-0037] Hans, Z. , Lee, D. B. , Zimmerman, M. A. , & Wiebe, D. J. (2025). Legacy of racism and firearm violence during the COVID‐19 pandemic in the United States. American Journal of Public Health, 115(2), 161–169. 10.2105/AJPH.2024.307891 39509679 PMC11715588

[ajcp70064-bib-0038] Hirschi, T. (1998). Social control theory: A control theory of delinquency, Criminology theory: Selected classic readings (2nd ed.). Routledge.

[ajcp70064-bib-0039] Hodgkinson, T. , Caputo, T. , & McIntyre, M. L. (2019). Beyond crime rates and community surveys: A new approach to police accountability and performance measurement. Crime Science, 8(1), 13. 10.1186/s40163-019-0108-x

[ajcp70064-bib-0040] Houghton, A. , Jackson‐Weaver, O. , Toraih, E. , Burley, N. , Byrne, T. , McGrew, P. , Duchesne, J. , Tatum, D. , & Taghavi, S. (2021). Firearm homicide mortality is influenced by structural racism in US metropolitan areas. Journal of Trauma and Acute Care Surgery, 91(1), 64–71. 10.1097/TA.0000000000003167 33797488

[ajcp70064-bib-0041] Jacobs, D. , & O'Brien, R. M. (1998). The determinants of deadly force: A structural analysis of police violence. American Journal of Sociology, 103(4), 837–862. 10.1086/231291

[ajcp70064-bib-0042] Jacoby, S. F. , Dong, B. , Beard, J. H. , Wiebe, D. J. , & Morrison, C. N. (2018). The enduring impact of historical and structural racism on urban violence in Philadelphia. Social Science & Medicine (1982), 199, 87–95. 10.1016/j.socscimed.2017.05.038 28579093 PMC7437144

[ajcp70064-bib-0043] Jones, C. P. (2000). Levels of racism: A theoretic framework and a gardener's tale. American Journal of Public Health, 90(8), 1212–1215. 10936998 10.2105/ajph.90.8.1212 PMC1446334

[ajcp70064-bib-0044] Jones, J. M. (1972). Prejudice and racism. Addison‐Wesley. https://cir.nii.ac.jp/crid/1130000796822130560

[ajcp70064-bib-0045] Kearney, M. S. , & Levine, P. B. (2014). Income inequality, social mobility, and the decision to drop out of high school (p. w20195). National Bureau of Economic Research. 10.3386/w20195

[ajcp70064-bib-0046] Knopov, A. , Rothman, E. F. , Cronin, S. W. , Franklin, L. , Cansever, A. , Potter, F. , Mesic, A. , Sharma, A. , Xuan, Z. , Siegel, M. , & Hemenway, D. (2019). The role of racial residential segregation in Black‐White disparities in firearm homicide at the state level in the United States, 1991–2015. Journal of the National Medical Association, 111(1), 62–75. 10.1016/j.jnma.2018.06.002 30129481

[ajcp70064-bib-0047] Krieger, N. (2012). Methods for the scientific study of discrimination and health: An ecosocial approach. American Journal of Public Health, 102(5), 936–944. 10.2105/AJPH.2011.300544 22420803 PMC3484783

[ajcp70064-bib-0048] Krieger, N. (2016). Living and dying at the crossroads: Racism, embodiment, and why theory is essential for a public health of consequence. American Journal of Public Health, 106(5), 832–833. 10.2105/AJPH.2016.303100 27049420 PMC4985119

[ajcp70064-bib-0049] Krieger, N. , Feldman, J. M. , Waterman, P. D. , Chen, J. T. , Coull, B. A. , & Hemenway, D. (2017). Local residential segregation matters: Stronger association of census tract compared to conventional city‐level measures with fatal and non‐fatal assaults (total and firearm related), using the index of concentration at the extremes (ICE) for racial, economic, and racialized economic segregation, massachusetts (US), 1995–2010. Journal of Urban Health: Bulletin of the New York Academy of Medicine, 94(2), 244–258. 10.1007/s11524-016-0116-z 28130678 PMC5391325

[ajcp70064-bib-0051] Larson, R. P. , Santaularia, N. J. , & Uggen, C. (2023). Temporal and spatial shifts in gun violence, before and after a historic police killing in Minneapolis. Spatial and Spatio‐Temporal Epidemiology, 47, 100602. 10.1016/j.sste.2023.100602 38042529 PMC10693656

[ajcp70064-bib-0052] Lee, D. B. , Hans, Z. , Aprill, S. L. , Stallworth, P. , Zimmerman, M. A. , Walton, M. A. , & Carter, P. M. (2025). Racialized economic segregation and youth firearm carriage: Community violence as a mediator. Journal of Behavioral Medicine, 48, 513–522. 10.1007/s10865-025-00564-z 40064764 PMC12927849

[ajcp70064-bib-0053] Lee, D. B. , Hsieh, H.‐F. , Stoddard, S. A. , Heinze, J. E. , Carter, P. M. , Goldstick, J. E. , Cunningham, M. C. , Cunningham, R. M. , & Zimmerman, M. A. (2020). Longitudinal pathway from violence exposure to firearm carriage among adolescents: The role of future expectation. Journal of Adolescence, 81(1), 101–113. 10.1016/j.adolescence.2020.03.009 32408115 PMC7325611

[ajcp70064-bib-0054] Lee, D. B. , Zimmerman, M. A. , Stallworth, P. , Cunningham, R. , Walton, M. , & Carter, P. M. (2024). Residential racial segregation and youth firearm aggression: Neighborhood disadvantage and exposure to violence as mediators. Youth & Society, 56, 1468–1490. 0044118X241256367 10.1177/0044118X241256367 40787609 PMC12333393

[ajcp70064-bib-0056] Leslie, T. F. , Frankenfeld, C. L. , & Hattery, A. J. (2022). Differentiating Black and hispanic: Outcome differences of segregated communities and police shootings in the USA, 2015–2020. Injury Epidemiology, 9(1), 8. 10.1186/s40621-022-00372-y 35241164 PMC8892749

[ajcp70064-bib-0057] Massey, D. S. , & Denton, N. A. (1998). American apartheid: Segregation and the making of the underclass. Harvard University Press. http://www.hup.harvard.edu/catalog.php?isbn=9780674018211

[ajcp70064-bib-0059] Mehranbod, C. A. , Gobaud, A. N. , Jacoby, S. F. , Uzzi, M. , Bushover, B. R. , & Morrison, C. N. (2022). Historical redlining and the epidemiology of present‐day firearm violence in the United States: A multi‐city analysis. Preventive Medicine, 165, 107207. 10.1016/j.ypmed.2022.107207 36027991 PMC10155117

[ajcp70064-bib-0060] Mesic, A. , Franklin, L. , Cansever, A. , Potter, F. , Sharma, A. , Knopov, A. , & Siegel, M. (2018). The relationship between structural racism and Black‐White disparities in fatal police shootings at the state level. Journal of the National Medical Association, 110(2), 106–116. 10.1016/j.jnma.2017.12.002 29580443

[ajcp70064-bib-0061] Meyer, I. H. (2003). Prejudice, social stress, and mental health in lesbian, gay, and bisexual populations: Conceptual issues and research evidence. Psychological Bulletin, 129(5), 674–697. 10.1037/0033-2909.129.5.674 12956539 PMC2072932

[ajcp70064-bib-0063] Neblett EW, J. r (2023). Racism measurement and influences, variations on scientific racism, and a vision. Social Science & Medicine (1982), 316, 115247. 10.1016/j.socscimed.2022.115247 36180279

[ajcp70064-bib-0064] Ousey, G. C. , & Augustine, M. C. (2001). Young guns: Examining alternative explanations of juvenile firearm homicide rates*. Criminology, 39(4), 933–968. 10.1111/j.1745-9125.2001.tb00945.x

[ajcp70064-bib-0065] Parks, N. , & Kirby, B. (2022). The function of the police force: A behavior‐analytic review of the history of how policing in america came to be. Behavior Analysis in Practice, 15(4), 1205–1212. 10.1007/s40617-021-00568-6 36605154 PMC9744977

[ajcp70064-bib-0066] Petrocelli, M. , Piquero, A. R. , & Smith, M. R. (2003). Conflict theory and racial profiling: An empirical analysis of police traffic stop data. Journal of criminal justice, 31(1), 1–11. 10.1016/S0047-2352(02)00195-2

[ajcp70064-bib-0067] Pettigrew, T. F. (1998). Intergroup contact theory. Annual Review of Psychology, 49(1), 65–85. 10.1146/annurev.psych.49.1.65 15012467

[ajcp70064-bib-0068] Poulson, M. , Neufeld, M. Y. , Dechert, T. , Allee, L. , & Kenzik, K. M. (2021). Historic redlining, structural racism, and firearm violence: A structural equation modeling approach. The Lancet Regional Health ‐ Americas, 3, 100052. 10.1016/j.lana.2021.100052 34888540 PMC8654098

[ajcp70064-bib-0069] Poulson, M. R. , Neufeld, M. Y. , LaRaja, A. , Allee, L. , Kenzik, K. M. , & Dechert, T. (2023a). Historic redlining, social mobility, and firearm violence. Journal of Trauma and Acute Care Surgery, 94(2), 312–319. 10.1097/TA.0000000000003757 35939375

[ajcp70064-bib-0070] Poulson, M. R. , Neufeld, M. Y. , Laraja, A. , Allee, L. , Kenzik, K. M. , & Dechert, T. (2023b). The effect of historic redlining on firearm violence. Journal of the National Medical Association, 115(4), 421–427. 10.1016/j.jnma.2023.06.003 37365061

[ajcp70064-bib-0071] Rafail, P. (2024). Community contexts predicting fatal police shootings of youth, 2014–2018. Youth & Society, 56(8), 1425–1444. 10.1177/0044118X241245145

[ajcp70064-bib-0072] Sampson, R. J. , & Groves, W. B. (1989). Community structure and crime: Testing social‐disorganization theory. American Journal of Sociology, 94(4), 774–802. 10.1086/229068

[ajcp70064-bib-0073] Sampson, R. J. , & Laub, J. H. (1997). A life‐course theory of cumulative disadvantage and the stability of delinquency, Developmental theories of crime and delinquency. Routledge

[ajcp70064-bib-0074] Schleimer, J. P. , Buggs, S. A. , McCort, C. D. , Pear, V. A. , Biasi, A. D. , Tomsich, E. , Shev, A. B. , Laqueur, H. S. , & Wintemute, G. J. (2022). Neighborhood racial and economic segregation and disparities in violence during the COVID‐19 pandemic. American Journal of Public Health, 112(1), 144–153. 10.2105/AJPH.2021.306540 34882429 PMC8713621

[ajcp70064-bib-0075] Shour, A. R. , Anguzu, R. , Zhou, Y. , Muehlbauer, A. , Joseph, A. , Oladebo, T. , Puthoff, D. , & Onitilo, A. A. (2023). Your neighborhood matters: An ecological social determinant study of the relationship between residential racial segregation and the risk of firearm fatalities. Injury Epidemiology, 10(1), 14. 10.1186/s40621-023-00425-w 36915201 PMC10012477

[ajcp70064-bib-0076] Siegel, M. , & Nicholson‐Robinson, V. (2025). Association between changes in racial residential and school segregation and trends in racial health disparities, 2000–2020: A life course perspective. Journal of Racial and Ethnic Health Disparities, 12(2), 1278–1297. 10.1007/s40615-024-01960-y 38421509 PMC11914365

[ajcp70064-bib-0077] Siegel, M. , Poulson, M. , Sangar, R. , & Jay, J. (2021). The interaction of race and place: predictors of fatal police shootings of Black victims at the incident, census tract, city, and state levels, 2013–2018. Race and Social Problems, 13(3), 245–265. 10.1007/s12552-020-09307-y

[ajcp70064-bib-0078] Siegel, M. , Rieders, M. , Rieders, H. , Moumneh, J. , Asfour, J. , Oh, J. , & Oh, S. (2023a). Measuring structural racism and its association with racial disparities in firearm homicide. Journal of Racial and Ethnic Health Disparities, 10(6), 3115–3130. 10.1007/s40615-022-01485-2 36508134 PMC9744051

[ajcp70064-bib-0079] Siegel, M. , Rieders, M. , Rieders, H. , Moumneh, J. , Asfour, J. , Oh, J. , & Oh, S. (2023b). Structural racism and racial health disparities at the state level: A latent variable approach. Journal of the National Medical Association, 115(4), 338–352. 10.1016/j.jnma.2023.07.003 37500328

[ajcp70064-bib-0080] Siegel, M. , Rieders, M. , Rieders, H. , Moumneh, J. , Asfour, J. , Oh, J. , & Oh, S. (2024). Using a latent variable method to develop a composite, multidimensional measure of structural racism at the city level. Journal of Racial and Ethnic Health Disparities, 11(4), 2271–2283. 10.1007/s40615-023-01695-2 37382871 PMC11236873

[ajcp70064-bib-0081] Siegel, M. , Sherman, R. , Li, C. , & Knopov, A. (2019). The relationship between racial residential segregation and Black‐White disparities in fatal police shootings at the city level, 2013–2017. Journal of the National Medical Association, 111(6), 580–587. 10.1016/j.jnma.2019.06.003 31256868

[ajcp70064-bib-0082] Sinyangwe, S. D. , McKesson, D. , & Elize, J. (2023). Mapping Police Violence Interactive Database. Mapping Police Violence Project. https://mappingpoliceviolence.org/

[ajcp70064-bib-0083] Smith, B. W. (2004). Structural and organizational predictors of homicide by police. Policing: An International Journal of Police Strategies & Management, 27(4), 539–557. 10.1108/13639510410566262

[ajcp70064-bib-0085] Spitzer, R. L. , Kroenke, K. , Williams, J. B. W. , & Löwe, B. (2006). A brief measure for assessing generalized anxiety disorder: The GAD‐7. Archives of Internal Medicine, 166(10), 1092–1097. 10.1001/archinte.166.10.1092 16717171

[ajcp70064-bib-0086] Stokely, C. , & Hamilton, C. V. (1967). Black power: The politics of liberation in America. Vintage

[ajcp70064-bib-0087] Stotzer, R. L. (2009). Violence against transgender people: A review of United States data. Aggression and Violent Behavior, 14(3), 170–179. 10.1016/j.avb.2009.01.006

[ajcp70064-bib-0088] Su, D. , Alshehri, K. , & Lawson, B. (2024). Association of racism experience with gun purchase during COVID‐19: Evidence from a national survey in the United States. Preventive Medicine Reports, 48, 102926. 10.1016/j.pmedr.2024.102926 39606099 PMC11600008

[ajcp70064-bib-0089] Trawalter, S. , Bart‐Plange, D.‐J. , & Hoffman, K. M. (2020). A socioecological psychology of racism: Making structures and history more visible. Current Opinion in Psychology, 32, 47–51. 10.1016/j.copsyc.2019.06.029 31377465

[ajcp70064-bib-0090] Uzzi, M. , Aune, K. T. , Marineau, L. , Jones, F. K. , Dean, L. T. , Jackson, J. W. , & Latkin, C. A. (2023). An intersectional analysis of historical and contemporary structural racism on non‐fatal shootings in Baltimore, Maryland. Injury Prevention: Journal of the International Society for Child and Adolescent Injury Prevention, 29, 85–90. 10.1136/ip-2022-044700 36301795 PMC9877125

[ajcp70064-bib-0091] Uzzi, M. , Whittaker, S. , Esposito, M. H. , Dean, L. T. , Buggs, S. A. , & Pollack Porter, K. M. (2024). Racial capitalism and firearm violence: Developing a theoretical framework for firearm violence research examining structural racism. Social Science & Medicine (1982), 358, 117255. 10.1016/j.socscimed.2024.117255 39197276 PMC11748318

[ajcp70064-bib-0093] Williams, D. R. , & Mohammed, S. A. (2013). Racism and health I: Pathways and scientific evidence. American Behavioral Scientist, 57(8), 1152–1173. 10.1177/0002764213487340 PMC3863357 24347666

[ajcp70064-bib-0094] Wirtz, A. L. , Poteat, T. C. , Malik, M. , & Glass, N. (2020). Gender‐based violence against transgender people in the United States: A call for research and programming. Trauma, violence & abuse, 21(2), 227–241. 10.1177/1524838018757749 29439615

[ajcp70064-bib-0095] Wong, B. , Bernstein, S. , Jay, J. , & Siegel, M. (2020). Differences in racial disparities in firearm homicide across cities: The role of racial residential segregation and gaps in structural disadvantage. Journal of the National Medical Association, 112(5), 518–530. 10.1016/j.jnma.2020.05.014 32641258

[ajcp70064-bib-0096] Wu, T.‐Y. , Hsieh, H.‐F. , Chow, C. M. , Yang, X. , Resnicow, K. , & Zimmerman, M. (2022). Examining racism and firearm‐related risks among Asian Americans in the United States during the COVID‐19 pandemic. Preventive Medicine Reports, 27, 101800. 10.1016/j.pmedr.2022.101800 35656206 PMC9152798

[ajcp70064-bib-0097] Wu, T.‐Y. , Hsieh, H.‐F. , Resnicow, K. , Carter, P. M. , Chow, C. M. , & Zimmerman, M. (2025). Understanding the intersectionality of COVID‐19 racism, mental distress, alcohol use, and firearm purchase behavior among Asian Americans. Journal of Racial and Ethnic Health Disparities, 12(1), 310–319. 10.1007/s40615-023-01874-1 38062320

[ajcp70064-bib-0098] Zaza, S. , Wright‐De Agüero, L. K. , Briss, P. A. , Truman, B. I. , Hopkins, D. P. , Hennessy, M. H. , Sosin, D. M. , Anderson, L. , Carande‐Kulis, V. G. , Teutsch, S. M. , & Pappaioanou, M. (2000). Data collection instrument and procedure for systematic reviews in the guide to community preventive services1. American Journal of Preventive Medicine, 18(1), 44–74. 10.1016/S0749-3797(99)00122-1. The names and affiliations of the Task Force members are listed on page v of this supplement and at http://www.thecommunityguide.org. 10806979

[ajcp70064-bib-0099] Zeimer, S. (2020). Exclusionary zoning, school segregation, and housing segregation: An investigation into a modern desegregation case and solutions to housing segregation note. Hastings Constitutional Law Quarterly, 48(1), 205–230.

[ajcp70064-bib-0100] Zimmerman, M. A. (2013). Resiliency theory: A Strengths‐Based approach to research and practice for adolescent health. Health Education & Behavior: The Official Publication of the Society for Public Health Education, 40(4), 381–383. 10.1177/1090198113493782 23863911 PMC3966565

