# Supplementary material for: Racism and racial disparities in firearm violence: A scoping review
Source: Am J Community Psychol. 2026 Apr 16;77(3-4):314–40. doi: 10.1002/ajcp.70064 (PMC13289513; doi:10.1002/ajcp.70064)
Supplement: Supplementary file 1 — Supporting File 1 [file AJCP-77-314-s001.docx]

**Appendix B**

Author(s):

Year of Publication:

Study Design:

Setting and Sample:

What is the Ecological-level of the study? Select all that apply:

- Individual-level
- Peer/family-level
- Community-level

Theoretically-driven research:

- Yes: Relevant theoretical model(s) are cited and discussed
- No Model: No theoretical model cited

If Yes: Relevant theoretical model(s) are cited and discussed:

Which theoretical model(s) was discussed?

Which racism concepts/measures was used?

Racism measurement breadth and depth:

- High: Multiple dimensions of racism evaluated (e.g., institutional, interpersonal)
- Moderate: One dimension of racism measured using multiple scales (e.g., institutional racism measured by redlining, racialized economic segregation measured by using ACS data)
- Low: One dimensions of racism measured using one scale (e.g., institutional racism measured by redlining only)

Firearm outcome(s) measured:

Firearm outcome measurement breadth and depth:

- High: Multiple types of firearm outcomes assessed using multiple sources of data (e.g., arrest data, self-report)
- Moderate_Type: Multiple types of firearm outcomes assessed using one data source (e.g., firearm carriage and firearm aggression assessed with arrest record)
- Moderate_Source: One type of firearm outcome assessed using multiple sources of data (e.g., firearm injury assessed with arrest and ED data)
- Low: One type of firearm outcome assessed with one data source

Highlight relevant findings (4 or more sentences):

Statistical tests:

- Yes: Statistical tests are appropriate given the research questions and data
- No: Statistical tests are NOT appropriate (e.g., does not account for clustering)

If No: Statistical tests are NOT appropriate (e.g., does not account for clustering) is selected:

If no, briefly describe why:

Any Moderators and/or Mediators:

- No effect modifiers or mediators
- Yes effect modifiers or mediators

If Any Moderators and/or Mediators: Yes effect modifiers or mediators is selected:

Please identify the moderators and/or mediators:

Is this a longitudinal study?

- Yes-longitudinal
- No-cross-sectional

Causal inference or correlation:

- High: Researchers estimate a causal effect and use appropriate methods (e.g., synthetic controls, propensity score matching, randomization, instrumental variables, etc)
- Moderate: Causal inference drawn, but methodology limits causal inference (e.g., groups not randomly assigned, but do not assess for/address selection bias)
- Low: Researchers estimate correlations

Probability-based sample

- No: Researchers used a non-probabilistic sampling approach - e.g., convenience sample, picked a particular city because firearm data was available there
- Yes: Researchers used a probabilistic sampling approach

Scope of sample

- High: National sample
- Moderate_Multi: Multiple states/cities/counties but not representative of US
- Moderate_Specific: State, city, or county wide analysis
- Low: Does not generalize to any region (state, city, town, country, county, etc)

Data recency

- High: Newer data only <5 years ago (from 2023)
- Moderate: Older and newer data (e.g., 2010-2020)
- Low: Older data only (>= 10 years ago)

Control variables? Please list:

Issues that limit your ability to interpret results (e.g., confounding variables not well controlled for, small N)? Briefly describe:
