# Supplementary material for: Racism and racial disparities in firearm violence: A scoping review
Source: Am J Community Psychol. 2026 Apr 16;77(3-4):314–40. doi: 10.1002/ajcp.70064 (PMC13289513; doi:10.1002/ajcp.70064)
Supplement: Supplementary file 2 — Supporting File 2 [file AJCP-77-314-s002.docx]

Appendix A: Boolean Search Terms

**Medline (OVID)**

Number of results: 563

1. Firearms/ or "gun violence"/ or "wounds, gunshot"/ or (gun or guns or rifle or rifles or pistol* or revolver* or semi-automatic* or shotgun* or ammunition or handgun* or firearm* or gunshot* or shooting* or "small arms").tw,kf. or (weapon* adj3 (carry* or carriage or purchas* or discharg* or owner* or owning)).tw,kf.

2. exp race relations/ or prejudice/ or exp racism/ or race factors/ or "social discrimination"/ or "social inclusion"/ or "social isolation"/ or "social segregation"/ or "social stigma"/ or stereotyping/ or racist.tw,kf. or racism.tw,kf. or anti-racis*.tw,kf. or antiracis*.tw,kf. or anti-black.tw,kf. or bigot*.tw,kf. or segregat*.tw,kf. or redlin*.tw,kf. or blockbust*.tw,kf. or ((race or races or racial* or ethno-rac*) adj10 (resentment* or stigma* or stereotyp* or relation* or discrim* or bias* or prejud* or oppress* or mistreat* or "unfair treat*" or exclusion or exclude* or deprivation or deprive* or disadvantag* or disinvest* or discriminat* or disparit* or unequal or equal* or equit* or incivil* or inequalit* or inequit* or intoleran* or marginaliz* or oppress*)).tw,kf. or ((institut* or policy or policies or historic* or structur* or systemic or systematic) adj10 (barrier* or "risk factor*" or cause or causes or competency or competencies)).tw,kf.

3. 1 and 2

**Scopus**

Number of results: 1740

1.

TITLE-ABS-KEY ( firearm* OR gun OR guns OR rifle OR rifles OR pistol* OR revolver* OR semi-automatic* OR shotgun* OR ammunition OR handgun* OR gunshot* OR shooting* OR "small arms" OR ( weapon* W/3 ( carry* OR carriage OR purchas* OR discharg* OR owner* OR owning ) ) )

2.

TITLE-ABS-KEY ( "Race relations" OR prejudice OR racism OR "race factors" OR "social discrimination" OR "social inclusion" OR "social isolation" OR "social segregation" OR "social stigma" OR stereotyping OR racist OR anti-racis* OR antiracis* OR anti-black OR bigot* OR redlin* OR blockbust* OR segregat* OR ( ( race OR races OR racial* OR ethno-rac* ) W/10 ( resentment* OR stigma* OR stereotyp* OR relation* OR discrim* OR bias* OR prejud* OR oppress* OR mistreat* OR "unfair treat*" OR exclusion OR exclude* OR deprivation OR deprive* OR disadvantag* OR disinvest* OR discriminat* OR disparit* OR unequal OR equal* OR equit* OR incivil* OR inequalit* OR inequit* OR intoleran* OR marginaliz* OR oppress* ) ) OR ( ( institut* OR policy OR policies OR historic* OR structur* OR systemic OR systematic ) W/10 ( barrier* OR "risk factor*" OR cause OR causes OR competency OR competencies ) ) )

3.

1 AND 2

**Cochrane**

Number of results: 18

1.

[mh Firearms] OR [mh "gun violence"] OR [mh "wounds, gunshot"] OR (gun:ti,ab OR guns:ti,ab OR rifle:ti,ab OR rifles:ti,ab OR pistol*:ti,ab OR revolver*:ti,ab OR semi-automatic*:ti,ab OR shotgun*:ti,ab OR ammunition:ti,ab OR handgun*:ti,ab OR firearm*:ti,ab OR gunshot*:ti,ab OR shooting*:ti,ab OR "small arms":ti,ab) OR (weapon*:ti,ab NEAR/3 (carry*:ti,ab OR carriage:ti,ab OR purchas*:ti,ab OR discharg*:ti,ab OR owner*:ti,ab OR owning:ti,ab))

2.

[mh "race relations"] OR [mh prejudice] OR [mh racism] OR [mh "race factors"] OR [mh "social discrimination"] OR [mh "social inclusion"] OR [mh "social isolation"] OR [mh "social segregation"] OR [mh "social stigma"] OR [mh stereotyping] OR racist:ti,ab OR racism:ti,ab OR anti-racis*:ti,ab OR antiracis*:ti,ab OR anti-black:ti,ab OR bigot*:ti,ab OR redlin*:ti,ab OR blockbust*:ti,ab OR segregat*:ti,ab OR ((race:ti,ab OR races:ti,ab OR racial*:ti,ab OR ethno-rac*:ti,ab) NEAR/10 (resentment*:ti,ab OR stigma*:ti,ab OR stereotyp*:ti,ab OR relation*:ti,ab OR discrim*:ti,ab OR bias*:ti,ab OR prejud*:ti,ab OR oppress*:ti,ab OR mistreat*:ti,ab OR ("unfair" NEXT treat*):ti,ab OR exclusion:ti,ab OR exclude*:ti,ab OR deprivation:ti,ab OR deprive*:ti,ab OR disadvantag*:ti,ab OR disinvest*:ti,ab OR discriminat*:ti,ab OR disparit*:ti,ab OR unequal:ti,ab OR equal*:ti,ab OR equit*:ti,ab OR incivil*:ti,ab OR inequalit*:ti,ab OR inequit*:ti,ab OR intoleran*:ti,ab OR marginaliz*:ti,ab OR oppress*:ti,ab)) OR ((institut*:ti,ab OR policy:ti,ab OR policies:ti,ab OR historic*:ti,ab OR structur*:ti,ab OR systemic:ti,ab OR systematic:ti,ab) NEAR/10 (barrier*:ti,ab OR ("risk" NEXT factor*):ti,ab OR cause:ti,ab OR causes:ti,ab OR competency:ti,ab OR competencies:ti,ab))

3.

(1 and 2)

**Embase**

Number of results: 357

1.

Firearm/exp OR 'gun violence'/de OR 'gunshot injury'/de OR (gun OR guns OR rifle OR rifles OR pistol* OR revolver* OR semi-automatic* OR shotgun* OR ammunition OR handgun* OR firearm* OR gunshot* OR shooting* OR 'small arms'):ti,ab OR (weapon* NEAR/3 (carry* OR carriage OR purchas* OR discharg* OR owner* OR owning)):ti,ab

2.

'race relation'/exp OR prejudice/de OR racism/exp OR 'race'/de OR ‘race difference’/de OR 'social discrimination'/de OR 'social inclusion'/de OR 'social isolation'/exp OR 'social stigma'/de OR stereotyping/de OR racist:ti,ab OR racism:ti,ab OR anti-racis*:ti,ab OR antiracis*:ti,ab OR anti-black:ti,ab OR bigot*:ti,ab OR redlin*:ti,ab OR blockbust*:ti,ab OR segregat*:ti,ab OR ((race OR races OR racial* OR ethno-rac*) NEAR/10 (resentment* OR stigma* OR stereotyp* OR relation* OR discrim* OR bias* OR prejud* OR oppress* OR mistreat* OR 'unfair treat*' OR exclusion OR exclude* OR deprivation OR deprive* OR disadvantag* OR disinvest* OR discriminat* OR disparit* OR unequal OR equal* OR equit* OR incivil* OR inequalit* OR inequit* OR intoleran* OR marginaliz* OR oppress*)):ti,ab OR ((institut* OR policy OR policies OR historic* OR structur* OR systemic OR systematic) NEAR/10 (barrier* OR 'risk factor*' OR cause OR causes OR competency OR competencies)):ti,ab

3.

([embase]/lim NOT ([embase]/lim AND [medline]/lim) OR [preprint]/lim)

4.

1 AND 2 AND 3

**PsycInfo (Ebsco)**

Number of results: 511

1.

(DE Firearms) OR (DE "gun violence") OR ((TI gun OR AB gun) OR (TI guns OR AB guns) OR (TI rifle OR AB rifle) OR (TI rifles OR AB rifles) OR (TI pistol* OR AB pistol*) OR (TI revolver* OR AB revolver*) OR (TI semi-automatic* OR AB semi-automatic*) OR (TI shotgun* OR AB shotgun*) OR (TI ammunition OR AB ammunition) OR (TI handgun* OR AB handgun*) OR (TI firearm* OR AB firearm*) OR (TI gunshot* OR AB gunshot*) OR (TI shooting* OR AB shooting*) OR (TI "small arms" OR AB "small arms")) OR ((TI weapon* OR AB weapon*) N3 ((TI carry* OR AB carry*) OR (TI carriage OR AB carriage) OR (TI purchas* OR AB purchas*) OR (TI discharg* OR AB discharg*) OR (TI owner* OR AB owner*) OR (TI owning OR AB owning)))

2.

DE "Racial and Ethnic Relations" OR DE "Interracial Interactions" OR DE "Racial and Ethnic Socialization" OR DE "Racial Bias" OR DE "Racial Privilege" OR (DE prejudice) OR DE "Race and Ethnic Discrimination" OR DE "Racism" OR DE "Internalized Racism" OR DE "Systemic Racism" OR (DE "social discrimination") OR (DE "social inclusion") OR (DE "social isolation") OR (DE stigma) OR (DE Stranger Reactions) OR (DE "Stereotyped Attitudes") OR (TI racist OR AB racist) OR (TI racism OR AB racism) OR (TI anti-racis* OR AB anti-racis*) OR (TI antiracis* OR AB antiracis*) OR (TI anti-black OR AB anti-black) OR (TI bigot* OR AB bigot*) OR (TI redlin* OR AB redlin*) OR (TI blockbust* OR AB blockbust*) OR (TI segregat* OR AB segregat*) OR (((TI race OR AB race) OR (TI races OR AB races) OR (TI racial* OR AB racial*) OR (TI ethno-rac* OR AB ethno-rac*)) N10 ((TI resentment* OR AB resentment*) OR (TI stigma* OR AB stigma*) OR (TI stereotyp* OR AB stereotyp*) OR (TI relation* OR AB relation*) OR (TI discrim* OR AB discrim*) OR (TI bias* OR AB bias*) OR (TI prejud* OR AB prejud*) OR (TI oppress* OR AB oppress*) OR (TI mistreat* OR AB mistreat*) OR (TI "unfair treat*" OR AB "unfair treat*") OR (TI exclusion OR AB exclusion) OR (TI exclude* OR AB exclude*) OR (TI deprivation OR AB deprivation) OR (TI deprive* OR AB deprive*) OR (TI disadvantag* OR AB disadvantag*) OR (TI disinvest* OR AB disinvest*) OR (TI discriminat* OR AB discriminat*) OR (TI disparit* OR AB disparit*) OR (TI unequal OR AB unequal) OR (TI equal* OR AB equal*) OR (TI equit* OR AB equit*) OR (TI incivil* OR AB incivil*) OR (TI inequalit* OR AB inequalit*) OR (TI inequit* OR AB inequit*) OR (TI intoleran* OR AB intoleran*) OR (TI marginaliz* OR AB marginaliz*) OR (TI oppress* OR AB oppress*))) OR (((TI institut* OR AB institut*) OR (TI policy OR AB policy) OR (TI policies OR AB policies) OR (TI historic* OR AB historic*) OR (TI structur* OR AB structur*) OR (TI systemic OR AB systemic) OR (TI systematic OR AB systematic)) N10 ((TI barrier* OR AB barrier*) OR (TI "risk factor*" OR AB "risk factor*") OR (TI cause OR AB cause) OR (TI causes OR AB causes) OR (TI competency OR AB competency) OR (TI competencies OR AB competencies)))

3.

1 AND 2

**Criminal Justice Abstracts (Ebsco)**

Number of results: 140

1.

TI ( Firearm* OR gun OR guns OR rifle OR rifles OR pistol* OR revolver* OR semi-automatic* OR shotgun* OR ammunition OR handgun* OR gunshot* OR shooting* OR "small arms" OR (weapon* N3 (carry* OR carriage OR purchas* OR discharg* OR owner* OR owning)) ) OR AB ( Firearm* OR gun OR guns OR rifle OR rifles OR pistol* OR revolver* OR semi-automatic* OR shotgun* OR ammunition OR handgun* OR gunshot* OR shooting* OR "small arms" OR (weapon* N3 (carry* OR carriage OR purchas* OR discharg* OR owner* OR owning)) ) OR SU ( Firearm* OR gun OR guns OR rifle OR rifles OR pistol* OR revolver* OR semi-automatic* OR shotgun* OR ammunition OR handgun* OR gunshot* OR shooting* OR "small arms" OR (weapon* N3 (carry* OR carriage OR purchas* OR discharg* OR owner* OR owning)) ) OR KW ( Firearm* OR gun OR guns OR rifle OR rifles OR pistol* OR revolver* OR semi-automatic* OR shotgun* OR ammunition OR handgun* OR gunshot* OR shooting* OR "small arms" OR (weapon* N3 (carry* OR carriage OR purchas* OR discharg* OR owner* OR owning)) )

2.

TI ( “Race relations” OR prejudice OR racism OR “race factors” OR "social discrimination" OR "social inclusion" OR "social isolation" OR "social segregation" OR "social stigma" OR stereotyping OR racist OR anti-racis* OR antiracis* OR anti-black OR bigot* OR segregat* OR ((race OR races OR racial* OR ethno-rac*) W/10 (stigma* OR stereotyp* OR relation* OR discrim* OR bias* OR prejud* OR oppress* OR mistreat* OR "unfair treat*" OR exclusion OR exclude* OR deprivation OR deprive* OR disadvantag* OR disinvest* OR discriminat* OR disparit* OR unequal OR equal* OR equit* OR incivil* OR inequalit* OR inequit* OR intoleran* OR marginaliz* OR oppress*)) OR ((institut* OR policy OR policies OR historic* OR structur* OR systemic) W/10 (barrier* OR "risk factor*" OR cause OR causes OR competency OR competencies)) ) OR KW ( “Race relations” OR prejudice OR racism OR “race factors” OR "social discrimination" OR "social inclusion" OR "social isolation" OR "social segregation" OR "social stigma" OR stereotyping OR racist OR anti-racis* OR antiracis* OR anti-black OR OR bigot* OR segregat* OR ((race OR races OR racial* OR ethno-rac*) W/10 (stigma* OR stereotyp* OR relation* OR discrim* OR bias* OR prejud* OR oppress* OR mistreat* OR "unfair treat*" OR exclusion OR exclude* OR deprivation OR deprive* OR disadvantag* OR disinvest* OR discriminat* OR disparit* OR unequal OR equal* OR equit* OR incivil* OR inequalit* OR inequit* OR intoleran* OR marginaliz* OR oppress*)) OR ((institut* OR policy OR policies OR historic* OR structur* OR systemic OR systematic) W/10 (barrier* OR "risk factor*" OR cause OR causes OR competency OR competencies)) ) OR SU ( “Race relations” OR prejudice OR racism OR “race factors” OR "social discrimination" OR "social inclusion" OR "social isolation" OR "social segregation" OR "social stigma" OR stereotyping OR racist OR anti-racis* OR antiracis* OR anti-black OR bigot* OR segregat* OR ((race OR races OR racial* OR ethno-rac*) W/10 (resentment* OR stigma* OR stereotyp* OR relation* OR discrim* OR bias* OR prejud* OR oppress* OR mistreat* OR "unfair treat*" OR exclusion OR exclude* OR deprivation OR deprive* OR disadvantag* OR disinvest* OR discriminat* OR disparit* OR unequal OR equal* OR equit* OR incivil* OR inequalit* OR inequit* OR intoleran* OR marginaliz* OR oppress*)) OR ((institut* OR policy OR policies OR historic* OR structur* OR systemic OR systematic) W/10 (barrier* OR "risk factor*" OR cause OR causes OR competency OR competencies)) ) OR AB(“Race relations” OR prejudice OR racism OR “race factors” OR "social discrimination" OR "social inclusion" OR "social isolation" OR "social segregation" OR "social stigma" OR stereotyping OR racist OR anti-racis* OR antiracis* OR anti-black OR bigot* OR segregat* OR ((race OR races OR racial* OR ethno-rac*) W/10 (resentment* OR stigma* OR stereotyp* OR relation* OR discrim* OR bias* OR prejud* OR oppress* OR mistreat* OR "unfair treat*" OR exclusion OR exclude* OR deprivation OR deprive* OR disadvantag* OR disinvest* OR discriminat* OR disparit* OR unequal OR equal* OR equit* OR incivil* OR inequalit* OR inequit* OR intoleran* OR marginaliz* OR oppress*)) OR ((institut* OR policy OR policies OR historic* OR structur* OR systemic OR systematic) W/10 (barrier* OR "risk factor*" OR cause OR causes OR competency OR competencies)) )

3.

1 AND 2

Sociological Abstracts (ProQuest)

Number of Results:

1.

MAINSUBJECT.EXACT("Firearms") OR title( (gun or guns or rifle or rifles or pistol* or revolver* or semi-automatic* or shotgun* or ammunition or handgun* or firearm* or gunshot* or shooting* or "small arms") or (weapon* NEAR/3 (carry* or carriage or purchas* or discharg* or owner* or owning)) OR ((gun or guns or rifle or rifles or pistol* or revolver* or semi-automatic* or shotgun* or ammunition or handgun* or firearm* or gunshot* or shooting* or "small arms") or (weapon* NEAR/3 (carry* or carriage or purchas* or discharg* or owner* or owning)))

) OR abstract( (gun or guns or rifle or rifles or pistol* or revolver* or semi-automatic* or shotgun* or ammunition or handgun* or firearm* or gunshot* or shooting* or "small arms") or (weapon* NEAR/3 (carry* or carriage or purchas* or discharg* or owner* or owning)) OR ((gun or guns or rifle or rifles or pistol* or revolver* or semi-automatic* or shotgun* or ammunition or handgun* or firearm* or gunshot* or shooting* or "small arms") or (weapon* NEAR/3 (carry* or carriage or purchas* or discharg* or owner* or owning)))

)

2.

(MAINSUBJECT.EXACT.EXPLODE("Racial Relations") OR MAINSUBJECT.EXACT("Prejudice") OR MAINSUBJECT.EXACT("Racism") OR MAINSUBJECT.EXACT("Social Isolation") OR MAINSUBJECT.EXACT.EXPLODE("Segregation") MAINSUBJECT.EXACT("Stigma") OR MAINSUBJECT.EXACT("Stereotypes")) OR title(racist OR racism OR anti-racis* OR antiracis* OR anti-black OR bigot* OR segregat* OR redlin* OR blockbust* OR ((race OR races OR racial* OR ethno-rac*) NEAR/10 (resentment* OR stigma* OR stereotyp* OR relation* OR discrim* OR bias* OR prejud* OR oppress* OR mistreat* OR "unfair treat*" OR exclusion OR exclude* OR deprivation OR deprive* OR disadvantag* OR disinvest* OR discriminat* OR disparit* OR unequal OR equal* OR equit* OR incivil* OR inequalit* OR inequit* OR intoleran* OR marginaliz* OR oppress*)) OR ((institut* OR policy OR policies OR historic* OR structur* OR systemic) NEAR/10 (barrier* OR "risk factor*" OR cause OR causes OR competency OR competencies))) OR abstract(racist OR racism OR anti-racis* OR antiracis* OR anti-black OR bigot* OR segregat* OR redlin* OR blockbust* OR ((race OR races OR racial* OR ethno-rac*) NEAR/10 (resentment* OR stigma* OR stereotyp* OR relation* OR discrim* OR bias* OR prejud* OR oppress* OR mistreat* OR "unfair treat*" OR exclusion OR exclude* OR deprivation OR deprive* OR disadvantag* OR disinvest* OR discriminat* OR disparit* OR unequal OR equal* OR equit* OR incivil* OR inequalit* OR inequit* OR intoleran* OR marginaliz* OR oppress*)) OR ((institut* OR policy OR policies OR historic* OR structur* OR systemic) NEAR/10 (barrier* OR "risk factor*" OR cause OR causes OR competency OR competencies)))

3. 1 and 2
